# Candidate gene biodosimetry markers of exposure to external ionizing radiation in human blood: A systematic review

**DOI:** 10.1371/journal.pone.0198851

**Published:** 2018-06-07

**Authors:** Jerome Lacombe, Chao Sima, Sally A. Amundson, Frederic Zenhausern

**Affiliations:** 1 Center for Applied NanoBioscience and Medicine, University of Arizona, Phoenix, Arizona, United States of America; 2 Center for Bioinformatics and Genomic Systems Engineering, Texas A&M Engineering Experiment Station, College Station, TX, United States of America; 3 Center for Radiological Research, Columbia University Medical Center, New York, NY, United States of America; 4 Honor Health Research Institute, Scottsdale, Arizona, United States of America; 5 Translational Genomics Research Institute, Phoenix, Arizona, United States of America; ENEA Centro Ricerche Casaccia, ITALY

## Abstract

**Purpose:**

To compile a list of genes that have been reported to be affected by external ionizing radiation (IR) and to assess their performance as candidate biomarkers for individual human radiation dosimetry.

**Methods:**

Eligible studies were identified through extensive searches of the online databases from 1978 to 2017. Original English-language publications of microarray studies assessing radiation-induced changes in gene expression levels in human blood after external IR were included. Genes identified in at least half of the selected studies were retained for bio-statistical analysis in order to evaluate their diagnostic ability.

**Results:**

24 studies met the criteria and were included in this study. Radiation-induced expression of 10,170 unique genes was identified and the 31 genes that have been identified in at least 50% of studies (12/24 studies) were selected for diagnostic power analysis. Twenty-seven genes showed a significant Spearman’s correlation with radiation dose. Individually, TNFSF4, FDXR, MYC, ZMAT3 and GADD45A provided the best discrimination of radiation dose < 2 Gy and dose ≥ 2 Gy according to according to their maximized Youden’s index (0.67, 0.55, 0.55, 0.55 and 0.53 respectively). Moreover, 12 combinations of three genes display an area under the Receiver Operating Curve (ROC) curve (AUC) = 1 reinforcing the concept of biomarker combinations instead of looking for an ideal and unique biomarker.

**Conclusion:**

Gene expression is a promising approach for radiation dosimetry assessment. A list of robust candidate biomarkers has been identified from analysis of the studies published to date, confirming for example the potential of well-known genes such as FDXR and TNFSF4 or highlighting other promising gene such as ZMAT3. However, heterogeneity in protocols and analysis methods will require additional studies to confirm these results.

## Introduction

A mass-casualty nuclear disaster, such as detonation of a terrorist dirty bomb or a nuclear power plant incident, requires an effective and fast planning for the medical response in order to treat and save thousands of lives. As such, there is a need to assess precisely the absorbed radiation dose for setting an effective triage of the affected population in order to distinguish those who need immediate medical intervention from those who are candidates for delayed treatment [1].

Professional radiation workers, astronauts or even patients wear a radiation detector, which can use a wide range of different physical and chemical interactions to convert dose to a directly measurable quantity, such as electronic charge collected from air ionization or color change arising from changes in atomic electronic states [2]. However, in the event of a radiological catastrophe, as the general population is not so equipped, dosimetry assessment cannot be performed with radiation detectors. Instead, it would be accomplished through a combination of physical dosimetry, history of an individual’s location, clinical signs and symptoms, and individual hematology assessment, with other methods such as the dicentric chromosome assay (DCA) used for long-term risk assessment [1].

Sullivan et al. detailed the different biological approaches for radiation dose assessment including DCA, gamma-H2AX foci assay, cytokinesis block micronucleus assay or “-omic” assays [1]. Although there is no biodosimetry method approved by the U.S. Food and Drug Administration (FDA) yet, the DCA is currently considered the “gold-standard”. This assay is very specific to IR and low background levels of dicentric chromosomes allow it to be highly sensitive. However, like all cytogenetics-based assays, the DCA is labor intensive and takes a long time to estimate the dose, an important limitation for radiation dose assessment in an emergency scenario. Indeed, early medical intervention has been shown to improve the survival of individuals after radiation exposure and some medical countermeasures are most effective when administered within the first 24 hours [1]. Alternative methods, such as the gamma-H2AX foci assay, electron paramagnetic resonance, or automation of pre-existing approaches are faster but require cost-intensive machines and large facilities [3–5].

The development of gene expression profiles, especially in peripheral blood lymphocytes, has been suggested as an alternate approach to radiation biodosimetry [6,7]. Exposure of human cells to environmental stresses, including IR, is known to activate multiple signal transduction pathways, and rapidly results in complex patterns of gene expression change. In contrast to DCA or the micronucleus assay, gene expression does not require cell division and can be analyzed quickly with advanced molecular assays. Moreover, recent improvements in microfluidics and “lab-on-chip” technology, may enable automation and miniaturization to provide a point-of-care device integrated in a high-throughput platform able to process and analyze large numbers of samples and return results in a few hours [8].

Several large-scale studies have investigated gene expression levels after irradiation. However, there is often a large discrepancy in the identified biomarkers and the reproducibility of results is unclear. The reasons for the observed variability may include different microarray platforms, variations in experimental protocols, and dissimilar statistical approaches.

The purpose of this paper was to conduct a systematic review of the scientific literature to compile a list of genes that have been reported to be affected by external ionizing radiation and to use their response level to assess them as candidates for individual human biodosimetry after IR exposure across the published studies. Blood is a preferred tissue for radiation biodosimetry both because white cells are highly radiation sensitive and show robust responses, and because collection is minimally invasive and can be performed in non-clinical settings [9]. As the great majority of gene expression biodosimetry studies have been performed using blood or blood cells, we focused our analysis on this model. Moreover, in order to avoid an “a-priori” selection, we focused our analysis on studies that did not use a candidate gene approach but performed large-scale screening to identify radiation-induced gene expression changes.

## Material and methods

### Literature search

We identified relevant studies using MEDLINE (1978–2017) and EMBASE (1990–2017) databases using the following search terms: *(“gene expression signature”[All Fields] OR “gene expression”[All Fields] OR “gene expression changes”[All Fields] OR “transcription response”[All Fields]) AND (“radiation exposure”[All Fields] OR “ionizing radiation” [All Fields] OR “radiotherapy”[All fields]) AND (“human peripheral blood”[All Fields] OR “human blood”[All Fields] OR “blood cells”[All Fields] OR “human cells”[All Fields]) AND/OR (“microarray” [All Fields])*. The search was conducted based on the Preferred Reporting Items for Systematic Reviews and Meta-analysis (PRISMA) guidelines [10] (S1 PRISMA Checklist). Two authors (JL and FZ) independently screened the titles and abstracts, with disagreements resolved by iteration and consensus. Where the suitability of the article was uncertain, the full text was assessed.

### Study eligibility

Papers were retained if they reported any large-scale approach (qRT-PCR has been excluded) to measure gene expression changes in human blood after external photonic ionizing radiation (ex-vivo or in-vivo). Studies on animal, plant or human cell lines were excluded as well as studies that investigated effects of ultraviolet radiation, electromagnetic field, internal emitters or particle radiation. Only studies published in English in peer-reviewed journals were included. Entries of review articles, conference abstracts, book chapters, editorials, or commentaries were excluded. We also excluded papers that did not provide a full list of radiation-modified gene expression with fold change and p-value, either in supplementary data or database. References from selected articles were also reviewed to ensure the inclusion of all relevant articles.

### Quality assessment and data extraction

Data extraction was performed using a standardised data extraction form (S1 File). We additionally used the Guidelines for the REporting of Tumor MARKer Studies (REMARK) to rank the selected publications and identified potential bias [11] (see S2 File for a detailed REMARK checklist). Because REMARK was created for oncology studies, we modified the criteria to enable use for radiation dosimetry studies. A collection of these modified REMARK (mREMARK) scores were then collated and ranked. REMARK scores between 15 and 20 were considered reflective of a higher quality study, with very low risk of bias. Studies with REMARK scores between 8 and 15 were considered to have a moderate risk of bias while those with REMARK scores below 8 were considered to have a high risk of bias and have been excluded from the analysis. Genes that have been reported in at least 50% of the selected studies (12 studies of 24) and whose expression is significantly altered (p<0.05) for at least one radiation dose and one time point have been included in this study.

### Statistics

A database has been created from raw data. For studies that did not provided fold changes for all gene, fold changes were calculated using the R package limma [12], after the raw data was normalized using loess normalization within the array and quantile normalization between the arrays. The Spearman’s rank correlation coefficient was used to assess dose dependence of the gene expression levels and test performance across all the studies. The individual gene biomarkers performance is based on Receiver Operating Characteristic (ROC) curves, which allow the characterization of the discrimination between two well-defined populations. The sensitivity, specificity, positive and negative predictive values of each gene were evaluated using the optimal threshold value calculated to maximize the Youden’s index. This index is defined as the sum of the sensitivity and specificity (expressed by a number comprised between 0 and 1) minus 1. All differences were considered statistically significant when p < 0.05. Analyses were performed with GraphPad Prism version 7.00 for Windows (GraphPad Software Inc., La Jolla, CA).

Additionally, the selected candidate genes were considered as the seed molecules from which we obtained direct and indirect protein-protein interactions using the STRING 10.5 database (Search Tool for the Retrieval of Interacting Genes) [13]. This database provides information on both experimental and predicted interactions from varied sources based on their neighborhood, gene fusions, co-occurrence, co-expression, experiments and literature mining. Additionally, the p53FamilyTargetGenes database (p53FamTaG), a comprehensive and reliable resource of genome-wide search of human p53, p63 and p73 direct target genes combining in silico prediction of p53 responsive elements (p53REs) has been used to identify p53 target genes [14].

## Results

### Literature search

**Fig 1:**
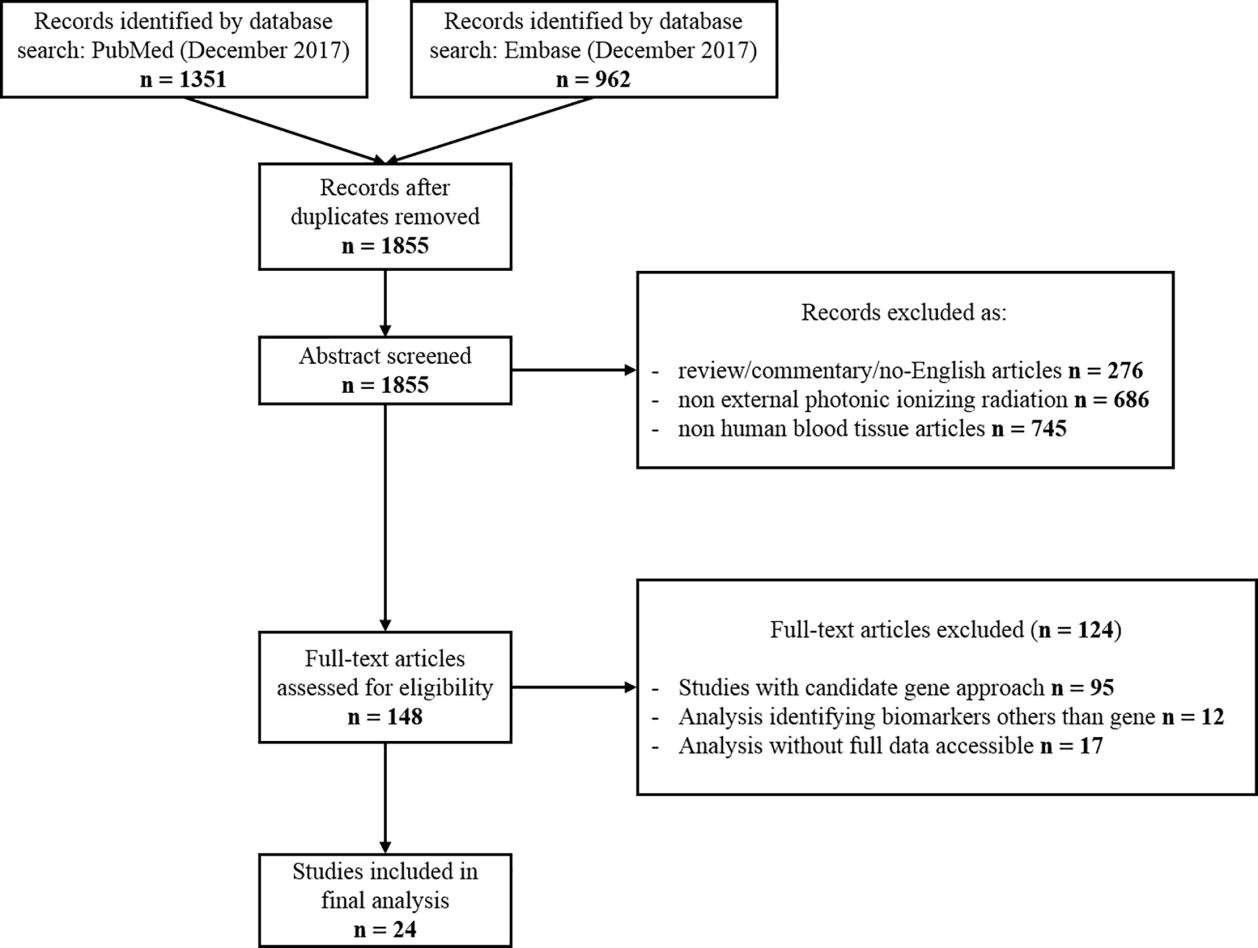
Flow diagram outlining the selection procedure to identify 24 articles that were included in the systematic review of gene radiation dosimetry biomarkers in human blood.

As shown in Fig 1, the initial search revealed a total of 2313 studies. After removing duplicates (n = 458), 1855 unique records were screened. After removing non-research articles and research papers that do not investigated effect of external photonic ionizing radiation on human blood tissue, 148 full-text articles were assessed for eligibility. Among these articles, we excluded 107 studies that did not use a large-scale approach or did not assess gene expression level. We also excluded 17 additional studies that did not provide full lists of gene expression changes with fold change and p-value of candidate genes or that provided unclear data analysis and insufficient details about presented results. Finally, data were extracted from 24 articles.

### Study characteristics and risk of bias

The characteristics of these 24 included studies are detailed in Table 1. Using the modified REMARK checklist to assess the risk of bias in the included studies, we found 7 with a low risk and 16 studies with a moderate risk. According to our criteria, one study has been found with a high risk [15] (S1 Table). However, this paper has not been excluded because it is a data paper and therefore, its low score is mainly due to the lack of data analysis and interpretation.

**Table 1:** Characteristics of included studies.

Study/Year	Donors (female/male)	IR source	Irradiated samples	RNA extraction source	Dose (Gy)	Dose-rate (Gy/min)	Analysis time after IR (h)	Analysis platform	Data accessibility
Beer et al. 2014. [21]	4 healthy donors (4M)	^137^Cs γ-Ray	PBMCs from heparinized WB in CellGro serum-free medium		60	NS	2, 4, 20	Agilent Whole Human Genome Oligo Microarray (8 × 60K; G4851A; #028004)	GEO database: GSE55955
Broustas et al. 2017. [22]	12 healthy donors (6F/6M)	X-Ray	WB in sodium citrate	WB diluted in RPMI-1640/10%FBS	0.1, 0.3, 0.5, 1, 2, 4	1.23	24	Agilent Whole Human Genome Microarrays v 2, 4x44K (G4112F)	GEO database: GSE90909
Dressman et al. 2007. [23]	27 patients with various diseases	NS	TBI	PBMCs	1.5–2	0.2	6	Custom human arrays printed at the Duke Microarray Facility using Operon Human Genome Oligo set (v. 3.0)	GEO database: GSE6874
El-Saghire et al. 2013. [24]	10 healthy donors	X-Ray	WB in EDTA	WB diluted in RPMI-1640/10%FBS	0.05, 1	0.03	8	GeneChip® Human Gene 1.0 ST Array	Selected genes available in S.M.
Fachin et al. 2007. [25]	4 healthy donors	^60^Co γ-Ray	PBMCs in RPMI-1640/20%FBS	PBMCs in RPMI-1640/20%FBS/2%PHA	0.1, 0.25, 0.5	1.18	48	Custom silanized glass side microarrays containing 4500 sequences from human IMAGE cDNA library Consortium	Selected genes available in S.M.
Ghandhi et al. 2015. [26]	5 healthy donors (2F/3M)	X-Ray	WB in sodium citrate diluted in RPMI-1640/10%FBS		0.56, 2.23, 4.45	0.0031, 1.03	24	Agilent Whole Human Genome Microarrays 4X44K v2 (G4112F)	GEO database: GSE65292
Gruel et al. 2008. [27]	5 healthy donors (5M)	^60^Co γ-Ray	WB in citrate-phosphate-dextrose diluted in RPMI-1640/10%FBS		0.05, 0.5	0.45	3, 24	Custom-built oligonucleotide microarrays	GEO database: GSE6978
Henríquez Hernández et al. 2009. [17]	24 BC patients	^60^Co γ-Ray	PBMCs from heparinized WB in cold DMEM/1% ultra-low-melting-point agarose	CD4+, CD8+, B and NK cells	2	1.5	24	Microarray containing 35.327 human 70-mer oligo probe sets (SweGene DNA Microarray Resource Center)	GEO database: GSE15341
Kabacik et al. 2011. [28]	3 healthy donors (3F)	X-Ray	WB		2, 4	0.7	2, 24	Breakthrough 20K cDNA microarray based on build 216 of the Unigene database.	Array Express database: E-TABM-1083
Knops et al. 2012. [29]	6 healthy donors (3F/3M)	^137^Cs γ-Ray	WB in heparin	PBMCs in RPMI-1640/10%FBS	0.02, 0.1, 0.5, 1, 2, 4	0.0286, 0.7	6, 24, 48	Agilent Whole Human Genome Microarrays v 2, 4x44K (G4112F)	Selected genes available in S.M.
Macaeva et al. 2016. [20]	10 healthy donors (5F/5M)	X-Ray	PBMCs from EDTA WB diluted in LGM-3		0.1, 1	0.26	8	GeneChip® Human Gene 1.0 ST Array	Selected genes available in S.M.
Mayer et al. 2011. [18]	24 H&N patients	^137^Cs γ-Ray	PBMCs from heparinized WB diluted in RPMI-1640/20%FBS		5	0.575	6	Illumina Human Sentrix-8 BeadChip arrays	Selected genes available in S.M.
Meadows et al. 2008. [30]	24 patients with various diseases	NS	TBI	PBMCs	1.5–2	0.2	6	Custom human arrays printed at the Duke Microarray Facility using Operon Human Genome Oligo set (v. 4.0)	GEO database: GSE10640
Nosel et al. 2013. [31]	5 healthy donors (5M)	^60^Co γ-Ray	WB in heparin diluted 1:10 in IMDM	CD4+ T lymphocytes	0.005, 0.01, 0.025, 0.05, 0.1, 0.5	0.05	2.5, 5, 7.5, 10	Agilent Whole Human Genome Microarrays v 2, 4x44K	GEO database: GSE43151
Paul & Amundson. 2008. [32]	10 healthy donors (5F/5M)	^137^Cs γ-Ray	WB in sodium citrate	WB diluted in RPMI-1640/10%FBS	0.5, 2, 5, 8	0.82	6, 24	Agilent Whole Human Genome Oligo Microarrays (G4112A)	GEO database: GSE8917
Paul & Amundson. 2011. [33]	24 healthy donors (12F/12M)	^137^Cs γ-Ray	WB in sodium citrate	WB diluted in RPMI-1640/10%FBS	0.1, 0.5, 2	0.82	6	Agilent Whole Human Genome Oligo Microarrays (G4112A)	GEO database: GSE23515
Paul et al. 2011. [34]	18 patients with various diseases	X-ray	TBI	WB in PAXgene RNA tubes	1.25, 3.75	0.1	4, 24	Agilent Whole Human Genome Oligo Microarrays (G4112A)	GEO database: GSE20162
Paul et al. 2013. [35]	5 healthy donors (3F/2M)	^137^Cs γ-Ray	WB in sodium citrate	WB diluted in RPMI-1640/10%FBS	0.5, 2, 5, 8	0.82	48	Agilent Whole Human Genome Oligo Microarrays (G4112A)	GEO database: GSE44201
Pogosova-Agadjanyan et al. 2011. [36]	8 healthy donors (4F/4M)	γ-Ray	Expanded T cells in X-VIVO 15/5%FBS		0.15, 12	7.6	3, 8, 24	Affimetrix GeneChip Human Genome U133 Plus 2.0 Arrays	Selected genes available in S.M.+ GEO database (accession number NS)
Rouchka et al. 2016. [15]	4 healthy donors	^137^Cs γ-Ray	WB in EDTA	WBCs	0.3, 1.5, 3	5.6	0.5	Affymetrix® Human Gene 1.0 ST v1 Arrays	GEO database: GSE64375
Templin et al. 2011. [37]	8 patients for stem cell transplation	X-ray	TBI	WB in PAXgene RNA tube	1.25	NS	4	Agilent Whole Human Genome Oligo Microarrays (G4112A)	GEO database: GSE23393
Versteyhe et al. 2013. [38]	8 healthy donors	^137^Cs γ-Ray	T-cells in RPMI-1640/10%FBS/2mM L-glutamine/1mM sodium pyruvate/0.05mM 2-mercaptoethanol		1	2	4	Affimetrix GeneChip Human Genome U133 Plus 2.0 Arrays	GEO database: GSE39156
Vinoth et al. 2014. [16]	Healthy donors	γ-Ray	PBMCs from heparinized WB diluted in RPMI-1640/10%FBS		1	1.16	0.5	Illumina HumanRef8 V3.0, Human Whole-Genome Expression BeadChips	GEO database: GSE36355
Wen et al. 2011. [39]	6 high risked ALL patients (3M/3F)	NS	TBI	PBMCs	4.5 & 9	0.045	24	Agilent Whole Human Genome Oligo Microarrays (G4112A)	Selected genes available in S.M.

ALL: acute lymphoblastic leukemia; BC: breast cancer; H&N: head and neck cancer; FBS: fetal bovine serum; GEO, Gene Expression Omnibus; IDMD: Iscove’s Modified Dulbecco’s Medium; IR: ionizing radiation; F: female; M: male; NS: non specified; PBMC, peripheral blood mononuclear cell; S.M., Supplementary Materials; TBI: total body irradiation; WB: whole blood; WBC: white blood cells

Most studies assessed the effects of gamma and X-ray irradiation on blood tissue from healthy individuals after ex-vivo irradiation. However, five of the studies, assessed gene expression change after total body irradiation (TBI), and therefore, recruited patients and not healthy individuals. The combined population of the twenty-four studies totaled 264 participants. Total sample size ranged from 45 to 386 patients. The median number of participants per study was 8 (range 3 to 27). In addition, Vinoth et al. [16] does not specify the number of participants and so, these have not been taken included in these numbers. The primary goal of two studies was not to investigate biodosimetry but to predict radiotoxicity [17,18]. However, these studies filled all our criteria as they assessed gene expression changes in blood by using a microarray approach after irradiation and therefore have been retained. All together, these studies investigated a large range of dose (from 0.005 to 60 Gy), dose-rate (from 3.1 mGy/min to 7.6 Gy/min) and time of analysis after irradiation (from 30 min to 48 h); however, radiation-induced responses are extremely time- and dose-dependent. Nevertheless, we did not restrain our study to a more specific time or dose range because of our desire to identify a unique biomarker signature able to discriminate any radiation dose exposure after any time. The sample preparation also differs widely, either in the type of irradiated samples, the source of RNA used, or the microarray platform. Indeed, most studies performed an *ex-vivo* irradiation directly on anticoagulated whole blood (WB) or after isolating peripheral blood mononuclear cells (PBMCs). Only five studies used *in vivo* irradiation, collecting blood samples from patients during the course of TBI. In the same way, some studies analyzed radiation-induced gene expression changes in WB (that included all white blood cells such as mononuclear and polynuclear cells) whereas other studies used a post-irradiation density gradient to select PBMCs and then analyzed changes only in this population. Although it appears that radiation induced gene expression may vary among lymphocyte subsets [19], there is a high degree of overlap in radiation responsive genes detected in peripheral blood and in isolated PBMCs [20]. Based on this, we have included studies using both WB and isolated PBMC in our analysis.

### Reported radiation dosimetry biomarkers and diagnostic ability

All together, these studies identified more than 10,000 unique genes with significant radiation-induced changes of expression level for at least one radiation dose. Among these, 41.9% are down-regulated and 58.1% up-regulated (S2 Table).

Biomarkers identified in only one microarray dataset are usually irreproducible across studies, even if the investigated samples have similar clinical parameters [40]. Thus, we decided for this review to select for analysis the genes whose expression was significantly altered after at least one radiation dose in at least 50% of studies (12 studies of 24). We list the 31 genes that have met these criteria in Table 2.

**Table 2:** Characteristics of the 31 selected genes.

Gene Symbol	Gene name	Biological process	Molecular function	p53 target (number of REs)	Expression	Number of studies	References
ACTA2	actin, alpha 2, smooth muscle, aorta	muscle contraction	ATP binding	Y (3)	↑	17	[16,18,20,22–24,26,28–37]
AEN*	apoptosis enhancing nuclease	apoptosis	exonuclease activity, nucleic acid binding	N	↑	19	[16,18,20–24,26,27,29–38]
ASCC3	activating signal cointegrator 1 complex subunit 3	cell proliferation, DNA repair	ATP binding	Y (2)	↑	17	[18,20–22,24–26,28–36,38]
BAX	BCL2 associated X, apoptosis regulator	apoptosis	chaperone binding	Y (3)	↑	20	[16,18,20–24,26–38]
BBC3	BCL2 binding component 3	apoptosis	protein binding	Y (2)	↑	17	[20–23,26–38]
CCNG1	cyclin G1	cell cycle regulation	cyclin	Y (3)	↑	17	[16,18,20,22,24–26,28,29,31–38]
CD70	CD70 molecule	apoptosis, cell-cell signaling	receptor binding	Y (1)	↑	15	[16,20,22,24,26,28,29,31–38]
CDKN1A	cyclin dependent kinase inhibitor 1A	cell cycle arrest, DNA damage response, apoptosis	cyclin binding	Y (7)	↑	17	[16–18,20–24,26,28,29,32–37]
DDB2	damage specific DNA binding protein 2	DNA repair	DNA binding	Y (11)	↑	21	[17,18,20–26,28–39]
EI24	EI24, autophagy associated transmembrane protein	apoptosis, autophagy	p53 binding	Y (5)	↑	14	[18,20,21,25,26,28–34,36,39]
FBXO22	F-box protein 22	proteasome-dependent degradation	ubiquitin-protein transferase activity	Y (9)	↑	13	[16,18,20,24,26,28,29,31–36]
FDXR	ferredoxin reductase	metabolism, oxidation-reduction process	ferredoxin-NADP+ reductase activity	Y (3)	↑	16	[18,20–22,24,26,27,29,31–38]
GADD45A	growth arrest and DNA damage inducible alpha	apoptosis, cell cycle arrest, DNA repair	kinase binding	Y (1)	↑	17	[16,18,20,22–24,26,28–37]
IER5	immediate early response 5	cell proliferation, response to heat	protein binding	Y (1)	↑	16	[16–18,21,22,26–29,31–37]
MDM2	MDM2 proto-oncogene	cellular response to stimulus	ubiquitin-protein ligase	Y (6)	↑	16	[17,18,20–22,24,26,28,29,31–36,38]
MYC	MYC proto-oncogene, bHLH transcription factor	cell cycle arrest	DNA binding	Y (2)	↓	16	[20–24,26,28,29,31–37,39]
PCNA	proliferating cell nuclear antigen	DNA repair, cell proliferation	DNA polymerase processivity factor	Y (1)	↑	21	[15,17,18,20–24,26–38]
PHPT1	phosphohistidine phosphatase 1	cell metabolism	ion channel binding	Y (1)	↑	14	[18,20–22,24,26,28,29,31–35,37]
PLK2	polo like kinase 2	DNA damage response	ATP binding, signal transducer activity	Y (3)	↑	13	[17,20–22,26,28,29,32–37]
POLH	polymerase (DNA) eta	DNA repair	DNA binding	Y (3)	↑	15	[18,20,22,24–26,28,29,31–35,37,38]
RPS27L	ribosomal protein S27 like	DNA repair, apoptosis	RNA binding	Y (1)	↑	17	[15,18,20–24,26–29,31–36]
SESN1	sestrin 1	oxidation-reduction process	peroxiredoxin activity	Y (4)	↑	18	[16,18,20–26,28–30,32–37]
TIGAR^$^	TP53 induced glycolysis regulatory phosphatase	apoptosis, autophagy	phosphatase activity	N	↑	13	[18,20–22,25,26,28,29,31,32,34,37,38]
TMEM30A	transmembrane protein 30A	transmembrane transport	protein binding	Y (3)	↑	16	[17,20,22–24,26,28–37]
TNFRSF10B	TNF receptor superfamily member 10b	apoptosis, immune response	receptor activity	Y (8)	↑	17	[16,18,20–22,24,26,27,29,31–38]
TNFSF4	TNF superfamily member 4	immune response	receptor binding	Y (5)	↑	13	[18,20,22,25,26,29,32–38]
TRIAP1	TP53 regulated inhibitor of apoptosis 1	apoptosis, DNA damage response	p53 binding	N	↑	19	[16,18,20–24,26,28–38]
TRIM22	tripartite motif containing 22	immune response	transcription factor activity	Y (3)	↑	17	[18,20,22–26,26,29–34,36–38]
XPC	XPC complex subunit, DNA damage recognition and repair factor	DNA repair	DNA binding	Y (5)	↑	18	[17,18,20,22–26,28–36,38]
ZMAT3	zinc finger matrin-type 3	apoptosis	RNA binding	N	↑	17	[16,20–24,26,27,29–36,38]
ZNF79	zinc finger protein 79	transcription	DNA binding	Y (3)	↑	13	[18,20,22,26,29,31–38]

N, No; REs, Response Elements; Y, Yes.

* alternative name: ISG20L1.

$ alternative name: C12orf5.

**Fig 2:**
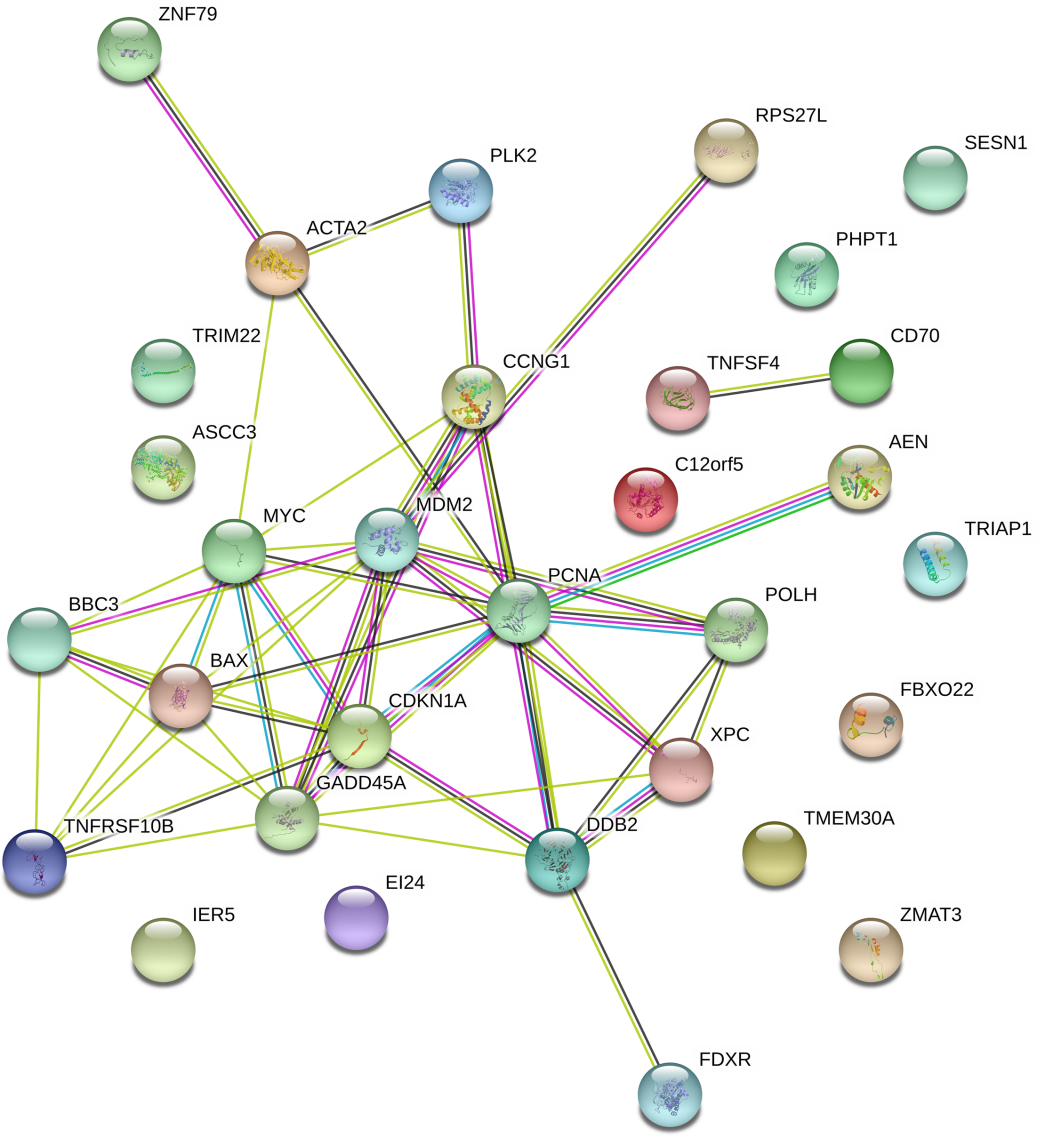
Protein-protein interaction enrichment network generated in STRING 10.5 using the 31 selected seed genes. The edges represent protein-protein association. Blue and purple edges are known interactions (from curated databases and experimentally determined, respectively). Yellow and black edges are interactions derived from text-mining and co-expression respectively. The green edge between AEN and PCNA is a predicted interaction as gene neighborhood.

DDB2 and PCNA are the genes both identified by the most studies (20/24) whereas FBXO22, PLK2, TIGAR, TNFSF4 and ZNF49 have only been found in thirteen studies. Interestingly, 97% of these genes (30/31) are up-regulated after IR and only the MYC gene is down-regulated. Not surprisingly, the enrichment analysis for Gene Ontologies shows that majority of these genes are involved in cellular responses to stress such as radiation, DNA damage stimulus, UV, etc. (S3 Table). The majority of these genes (27/31) are known to be directly regulated by p53, meaning these genes contain response elements (RE) that are recognized by sequence-specific nuclear transcription factors encoding by p53. However, although AEN, TIGAR, TRIAP and ZMAT3 are not identified as having classic p53 RE, they are known to be p53 inducible [41–44]. Moreover, the extended protein-protein interaction network generated using the 31 seed genes in STRING resulted in 51 interactions, whereas only 12 interactions were expected (Fig 2). This means that there are more interactions among the proteins than what would be expected for a random set of seed genes of similar size, suggesting that the entities are at least partially biologically connected, as a group.

In order to select genes that could be good candidate radiation dosimetry biomarkers, we calculated the Spearman's rank correlation coefficient by using the radiation dose and fold change from the raw data of the different included studies. For this, we decided to focus on radiation doses between 1 and 8 Gy. Human acute radiation syndrome (ARS) manifests following whole-body or partial-body irradiation in humans at estimated radiation doses generally above 1 Gy and delivered at relatively high dose rates. Individuals receiving doses < 1 Gy may still require medical management and treatment for symptoms, but delay of treatment could be considered since the level of exposure is not expected to pose immediate danger to life, allowing for judicious use of scarce resources in a radiation mass casualty incident. The LD_50_ (50% lethal dose) radiation dose for humans is estimated between 3.5 and 4.5 Gy but with increasing levels and qualities of supportive care, the estimated range of the LD_50_ values increases to 6–8 Gy with clinically unmanageable gastrointestinal syndrome dominating above 8–10 Gy. As the current strategies for mitigation are focused on the hematopoietic syndrome, we decided to exclude from the statistical analysis all data points with a dose > 8 Gy. Results showed that the expression of 27/31 genes is significantly correlated to the radiation dose within the 1–8 Gy range (S1 Fig). Only EI24, BBC3, BAX and TIGAR do not display a significant correlation with the radiation dose. TNSF4 has the highest Spearman’s rank correlation coefficient (r = 0.73). The majority of genes showed a significant increasing monotonic trend between their expression and the radiation dose, while the MYC gene showed a decreasing monotonic trend (Table 3).

**Table 3:** Genes classified according to their Spearman’s rank correlation coefficient (r) and p-value between their normalized expression level and radiation dose across all 24 studies.

Gene	R	p-value
TNFSF4	0.7305	<0.0001
TMEM30A	0.5439	<0.0001
ZMAT3	0.5413	<0.0001
FDXR	0.4641	<0.0001
AEN	0.4361	0.0002
MDM2	0.4617	0.0003
PCNA	0.413	0.0007
MYC	-0.4608	0.0011
ZNF79	0.4477	0.0011
PLK2	0.4632	0.0014
TRIAP1	0.3944	0.0018
GADD45A	0.3975	0.002
CD70	0.3816	0.0044
ACTA2	0.399	0.0045
DDB2	0.3269	0.0051
XPC	0.3482	0.0052
CDKN1A	0.3857	0.0057
TNFRSF10B	0.3404	0.0068
ASCC3	0.3585	0.0084
TRIM22	0.3454	0.014
FBXO22	0.3458	0.0186
IER5	0.37	0.0188
SESN1	0.3224	0.0224
POLH	0.2952	0.0245
CCNG1	0.2846	0.0289
RPS27L	0.2556	0.0415
PHPT1	0.2592	0.0456
EI24	0.2735	0.1014
BBC3	0.1677	0.2002
BAX	0.1342	0.2983
TIGAR	0.08259	0.6031

In a case of radiological incident, the technical requirements for initial triage include that it be accurate enough to identify anyone with a dose above 2 Gy for consideration of urgent treatment for ARS (although this 2 Gy threshold might vary to fit special populations, injured individuals or available resources) [45]. Therefore, in order to assess the diagnostic ability of the selected genes, we performed ROC curve analysis to discriminate radiation doses below 2 Gy from doses equal to or above 2 Gy as shown in S2 Fig. Candidate genes are able to discriminate these two groups with AUCs ranging from 0.507 (TIGAR) to 0.860 (TNFSF4) (Table 4). Only CD70 gene show a specificity of 100% for a sensitivity of 52%. Among the five genes with the highest Youden’s index (TNFSF4, FDXR, MYC, ZMAT3 and GADD45A), all of them display at least a sensitivity superior to 55% and a specificity superior to 80%. It is interesting to note that TNFSF4 which has the highest Youden’s index (0.67) is also the one with the highest Spearman’s rank correlation coefficient.

**Table 4:** AUC and diagnostic accuracies of the 33 genes discriminating radiation dose <2Gy and ≥2Gy classified according to their maximized Youden’s index.

Gene	AUC [95% CI]	Sensitivity (%)	Specificity (%)	PPV (%)	NPV (%)	False positive (%)	False negative (%)	Youden's index
TNFSF4	0.860, [0.75–0.97]	75	92	90	79	10	21	0.67
FDXR	0.742, [0.60–0.88]	75	80	69	85	31	15	0.55
MYC	0.678, [0.51–0.84]	60	95	94	68	6	32	0.55
ZMAT3	0.776, [0.64–0.91]	59	96	93	74	7	26	0.55
GADD45A	0.641, [0.49–0.79]	57	96	94	68	6	33	0.53
CD70	0.672, [0.52–0.83]	52	100	100	68	0	33	0.52
AEN	0.700, [0.55–0.85]	62	89	76	80	24	20	0.5
TNFRSF10B	0.650, [0.49–0.81]	73	75	62	83	38	17	0.48
ZNF79	0.734, [0.59–0.88]	60	88	83	69	17	31	0.48
PCNA	0.661, [0.52–0.80]	50	97	94	66	6	34	0.47
TRIAP1	0.648, [0.50–0.80]	57	90	85	68	15	33	0.47
TMEM30A	0.683, [0.54–0.83]	64	82	78	70	22	30	0.46
ACTA2	0.676, [0.52–0.83]	50	95	93	59	7	41	0.45
MDM2	0.684, [0.54–0.83]	52	93	88	65	12	35	0.45
PLK2	0.643, [0.48–0.81]	56	89	88	57	12	43	0.44
TRIM22	0.656, [0.49–0.82]	57	86	75	74	25	26	0.43
XPC	0.644, [0.50–0.79]	57	86	76	71	24	29	0.43
CCNG1	0.613, [0.45–0.77]	54	88	78	71	22	29	0.42
DDB2	0.610, [0.46–0.76]	47	95	88	69	12	31	0.42
PHPT1	0.592, [0.43–0.75]	61	81	74	70	26	30	0.42
RPS27L	0.591, [0.44–0.75]	59	82	74	70	26	30	0.41
ASCC3	0.636, [0.48–0.79]	52	88	82	64	18	36	0.4
IER5	0.628, [0.44–0.81]	68	72	75	65	25	35	0.4
CDKN1A	0.625, [0.47–0.78]	52	86	83	56	17	44	0.37
EI24	0.537, [0.35–0.73]	53	83	77	63	23	38	0.36
POLH	0.603, [0.45–0.75]	46	90	81	64	19	36	0.36
BBC3	0.572, [0.42–0.73]	56	79	68	68	32	32	0.34
FBXO22	0.627, [0.46–0.79]	62	70	73	58	27	42	0.32
SESN1	0.611, [0.45–0.77]	40	88	77	59	23	41	0.28
BAX	0.512, [0.36–0.66]	27	94	80	58	20	42	0.2
TIGAR	0.507, [0.32–0.69]	16	100	100	59	0	41	0.16

AUC: Area Under the Curve; CI: Confidence Interval; NPV: Negative Predictive Value; PPV: Positive Predictive Value

As sampling time may play an important role in biodosimetry studies and in the performance of potential biomarkers, we also performed an additional analysis comparing gene performance between early time (≤ 6 hours) and long time (≥ 24 hours) after irradiation by using spearman’s rank correlation coefficient (S4 Table) and AUC and diagnostic accuracies (S5 Table). First, results showed that few genes (ZMAT3, TNFSF4 and TMEM30) are significantly correlated to the radiation dose for time ≤ 6hours. Conversely, with the exception of BAX, all the genes showed a significant monotonic trend between their expression and the radiation dose for time ≥ 24 hours. Most of the included studies assessed gene expression for late (or middle) time points and therefore, few data points are available for early timing points and this can explain the poor significance for time ≤ 6 hours. However, these results showed high disparity between genes. For example, PLK2 or IER5 display a good correlation with radiation dose (r = 0.6041, p<0.0002 and r = 0.6881, p<0.0003 respectively) for time ≥ 24 hours whereas there is no correlation with time ≤ 6 hours (r = 0.068, p = 0.87 and r = 0.2242, p = 0.44 respectively). Conversely, although they do not display a significant correlation for early time point, some genes seem to be more consistent with later time (ZMAT3, TNFSF4, TMEM30A).

Interestingly, ROC curve analysis showed the same results profile with genes displaying a lower diagnostic performance for early time points compared to late time points. Indeed, twelve genes have Youden’s index ≥ 0.5 for time ≤ 6 hours whereas only two genes (TIGAR and BAX) have a Youden’s index <0.5 for time ≥ 24 hours. Candidate genes are able to discriminate dose ≤ 2 Gy and > 2 Gy with AUCs ranging from 0.514 (CD70) to 0.933 (ZMAT3) for time ≤ 6 hours and with AUCs ranging from 0.613 (BAX) to 0.904 (TNFSF4) for time ≥ 24 hours.

In a case of accidental radiation exposure and population triage, the intent may be to minimize misidentifying anyone as being below the threshold (‘false negative assignment’) so that very few people who could benefit are overlooked for receiving treatment [45]. Therefore, we calculated the diagnostic power of the 31 genes by setting a sensitivity of 100% in order to eliminate all false negatives (Table 5). Not surprisingly, the false positive rate increased drastically as specificity decreased. Seventeen genes even showed a specificity of 0%. TNFSF4, ZNF79, ZMAT3, FDXR and TMEM30A are the top 5 genes in this analysis and display specificity of 38, 24, 23, 17 and 14% respectively. With these settings, TNFSF4, FDXR and ZMAT3 are still in the top 5 genes. However, some genes like SESN1 or XPC ranked in the top 10 when sensitivity is fixed at 100% whereas they displayed bad performance compared to others genes when diagnostic accuracy was calculated according to the maximized Youden’s index. This suggests the interest of investigating an approach combining several biomarkers instead of looking at individual candidate genes in order to increase both specificity and sensitivity.

**Table 5:** Estimated specificity, positive predictive value rate, false positive and Youden’s index of the 33 genes discriminating radiation dose <2Gy and ≥2Gy at fixed sensitivity of 100%.

Gene	Specificity (%)	PPV (%)	False positive (%)	Youden's index
TNFSF4	38	62	38	0.38
ZNF79	24	57	43	0.24
ZMAT3	23	52	48	0.23
FDXR	17	41	59	0.17
TMEM30A	14	54	46	0.14
SESN1	12	53	47	0.12
TRIM22	10	45	55	0.1
XPC	9	47	53	0.09
TNFRSF10B	8	37	63	0.08
AEN	7	39	61	0.07
POLH	7	50	50	0.07
ACTA2	5	58	42	0.05
FBXO22	5	58	42	0.05
CCNG1	3	45	55	0.03
ASCC3	0	49	51	0
BAX	0	48	52	0
BBC3	0	44	56	0
CD70	0	48	52	0
CDKN1A	0	58	42	0
DDB2	0	44	56	0
EI24	0	50	50	0
GADD45A	0	51	49	0
IER5	0	54	46	0
MDM2	0	50	50	0
MYC	0	52	48	0
PCNA	0	49	51	0
PHPT1	0	46	54	0
PLK2	0	59	41	0
RPS27L	0	45	55	0
TIGAR	0	44	56	0
TRIAP1	0	49	51	0

PPV: Positive Predictive Value

**Fig 3:**
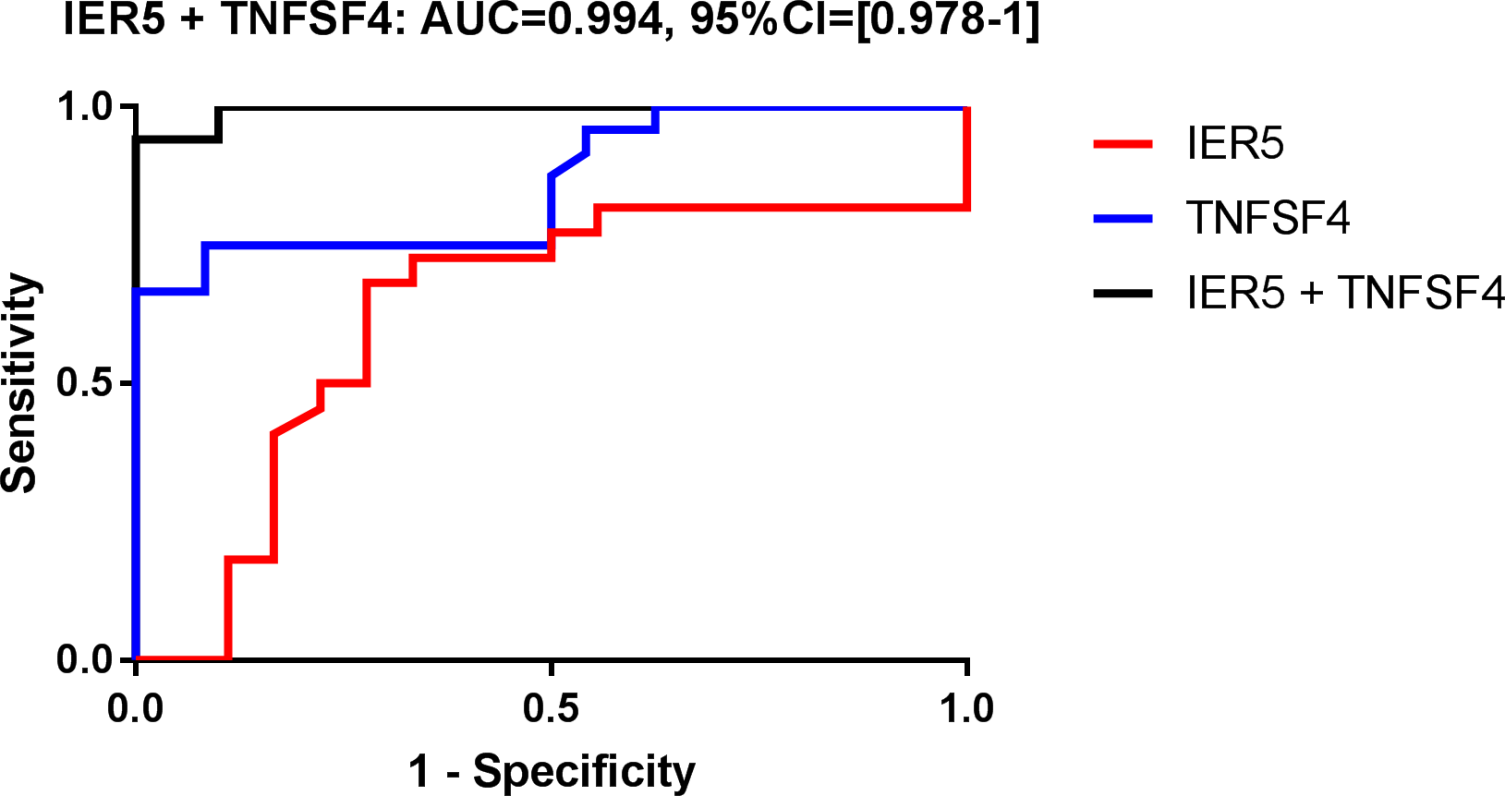
Receiver operating characteristic (ROC) curve analysis of IER5 + TNFSF4 combination that displays area under the ROC curve (AUC) ≥ 0.99 to discriminate radiation dose < 2 Gy from radiation dose ≥ 2 Gy.

Therefore, diagnostic power using gene combinations for these two groups was next assessed by first fitting a multiple linear regression model, and as in the individual gene analysis, plotting ROC curves and calculating AUCs using the model fitted values. When two genes are combined, four combinations showed an AUC superior to 0.95 (IER5+ZMAT3, CCNG1+TNFSF4, TNFSF4+TRIM22, BAX+TNFSF4 and IER5+TNFSF4). All of them include ZMAT3 or TNFSF4 that are in the top 3 with best AUC as individual markers when sensitivity is fixed at 100% (S3 Fig). However, the best combination of two genes for classification performance was obtained with the association of IER5 and TNFSF4 that showed an AUC = 0.994 (Fig 3). When three genes are combined, our statistical analysis showed that 12 combinations display an AUC = 1 (S6 Table).

### Particle irradiation

We only selected for this work the studies that assessed gene expression changes after photon irradiation (X-ray or γ-ray irradiation). However, in the case of a radiobiological event, irradiation may be different and may involve particles such as neutrons, protons or alpha particles. Therefore, there is also a need to identify radiation biomarkers for these specific types of irradiation. Although a few studies have assessed gene expression changes after particle irradiation in whole blood, especially by using a microarray approach, we identified three studies during our investigation that we report in Table 6. Unsurprisingly, many of the same genes respond to both X rays and particles. Indeed, among the 33 genes differentially regulated by photon irradiation, twenty-seven have also showed differential expression after particle irradiation in at least 2 of the 3 studies reported in Table 6. Three genes (BBC3, CD70 and MDM2) are even represented in all three studies. However, as raised by Broustas et al., the fold-change observed in response to neutrons is generally greater than the same dose of X rays [22].

**Table 6:** Large-scale studies assessing gene expression changes in human blood after particle irradiation.

Study/Year	Donors (female/male)	IR source	Irradiated samples	RNA extraction source	Dose (Gy)	Dose-rate (Gy/min)	Analysis time after IR (h)	Numbers of differentially expressed genes	Analysis platform	Data accessibility	Common differentially expressed genes between particle and photon irradiation
Broustas et al. 2017. [22]	12 healthy donors (6F/6M)	neutron	WB in sodium citrate	WB diluted in RPMI-1640/10%FBS	0.1	0.026	24	47	Agilent Whole Human Genome Microarrays v 2, 4x44K (G4112F)	GEO database: GSE90909	ACTA2, AEN, ASCC3, BAX, BBC3, CCNG1, CD70, CDKN1A, DDB2, FBXO22, FDXR, GADD45A, IER5, MDM2, MYC, PCNA, PHPT1, PLK2, POLH, RPS27L, SESN1, TIGAR, TMEM30A, TNFRSF10B, TNFSF4, TRIAP1, TRIM22, XPC, ZMAT3, ZNF79
					0.3			124			
					0.5			415			
					1			618			
Chauhan et al. 2014. [46]	12 healthy donors (6F/6M)	241Am α-particle	PBMCs in RPMI-1640/10%FBS/2mM L-glut/1%PS		0.5	0.98	24	30	Illumina human-12 v2 RNA BeadChips.	Selected genes available in main article	ACTA2, AEN, ASCC3, BAX, BBC3, CCNG1, CD70, CDKN1A, DDB2, FBXO22, FDXR, GADD45A, IER5, MDM2, PCNA, PHPT1, POLH, RPS27L, SESN1, TMEM30A, TNFRSF10B, TNFSF4, TRIAP1, TRIM22, XPC, ZNF79
					1			69			
					1.5			137			
Turtoi et al. 2010. [47]	2 healthy donors (1F/1M)	211At α-particle	PBMCs	PBMCs	0.05, 0.1, 0.2, 0.4, 0.8 & 1.6	NS	1h	338	Whole Human Genome 44K microarrays	Selected genes available in main article	BBC3, CD70, MDM2

FBS: fetal bovine serum; GEO, Gene Expression Omnibus; IR: ionizing radiation; F: female; M: male; PBMCs, peripheral blood mononuclear cells; WB: whole blood

### Mouse model

To evaluate radiation-induced changes of gene expression in humans, models are unfortunately limited. We saw that most studies used ex-vivo irradiation as a source of radiation. Obviously, this approach does not fit perfectly with the real situation encountered during an accidental radiation exposure since the blood cells are not exposed to internal stress signals that would be detected by circulating blood cells. Human *in vivo* radiation exposure may be studied by collecting blood samples from patients that receive radiation treatment. However, this model is still limited: patients are generally subjected to a fractionated radiation dose targeted to the tumor, limiting the dose to the blood. In order to study a range of total absorbed doses to the blood, it is thus necessary to collect samples after several fractions of treatment, but gene expression may not then reflect the effect of a single high radiation dose like during an acute accidental exposure event. Finally, there may be a lot of confounding factors as these patients almost always receive other systemic treatments such as chemotherapy or immunotherapy, or even may develop radiotoxicity, that can also modify gene expression. Given these constraints, the best human in vivo model is most likely patients undergoing total body irradiation but these samples may still be difficult to access. Finally, cancer patients may not be an appropriate model for the response of healthy individuals, as it is not fully understood how different disease states modify the baseline gene expression, or the radiation response.

For these reasons, much biodosimetry development has relied on animal models, especially mice, to allow in vivo irradiation to be performed in a completely controlled environment. However, nothing indicates that the gene radiation response will be identical in mouse compared to the human body. During our literature search, we identified several studies investigating radiation-induced changes of gene expression in mouse blood (Table 7). All these studies used C57BL/6 mice. We do not discuss the choice of this strain here, but it is important to note that the primary radiation response to DNA damage is strain dependent [48]. As variations in individual radiosensitivity exist in human, such differences are also present between mouse strains, highlighting the importance of population selection for experimental design. For example, several studies have reported the number of gamma-H2AX foci in skin or in blood lymphocytes following total body irradiation exposure differ and is higher in BALB/cJ mice than C57BL/6J [49,50].

**Table 7:** Large-scale studies assessing gene expression changes in mouse blood after irradiation.

Study/Year	Samples	IR source	Irradiated samples	RNA extraction source	Dose (Gy)	Dose-rate (Gy/min)	Analysis time after IR (days)	Analysis platform	Data accessibility	Numbers of differentially expressed genes	Common differentially expressed genes with human studies
Broustas et al. 2017. [52]	12 male C57BL/6 mice for each treatment	X-Ray	TBI	WB in PAXgene RNA tubes	1	0.007	1 & 7	Agilent Whole Mouse Genome Microarrays 4X44K v2 (G4846A)	GEO database: GSE85323	765 (1 day)—563 (7 days)	ACTA2, AEN, BAX, BBC3, CCNG1, CDKN1A, DDB2, EI24, FDXR, IER5, PCNA, PHPT1, PLK2, RPS27L, SESN1, TNFRSF10B, ZMAT3
					4					3577 (1 day)—1585 (7 days)	
		neutron			0.25	0.026				46 (1 day)—691 (7 days)	
					1					1956 (1 day)—6226 (7 days)	
Dressman et al. 2007. [23]	7 female C57BL/6 mice per group	^137^Cs γ-Ray	TBI	PBMCs	0.5, 2 & 10	4.8	6 hours	Custom mouse microarrays using Operon’s Mouse Genome Oligo set (v. 3.0)	GEO database: GSE6874	2,213	BAX, CDKN1A, EI24
Ghandhi et al. 2015. [53]	8 C57BL/6 mice per group	^90^Sr	Internal emitter	WB in PAXgene RNA tubes	1.2	0.3 Gy/day	4	Agilent Whole Mouse Genome Microarrays 4X44K v2 (G4846A)	GEO database: GSE64775	3957	CDKN1A, DDB2, FBXO22, FDXR, MYC, PHPT1, RPS24L, SESN1
					1.8	0.2 Gy/day	7			2633	
					2.1	0.15 Gy/day	9			3122	
					4.8	0.17 Gy/day	25			2683	
					5.3	0.1 Gy/day	30			4431	
Hyduke et al. 2013. [54]	male C57BL/6N mice	^137^Cs γ-Ray	TBI	WB in PAXgene RNA tubes	2 & 8	NS	6 hours	Agilent Whole Mouse Genome Microarrays (G4122F)	GEO database: GSE33172	NS	AEN, BAX, BBC3, CCNG1, CDKN1A, EI24, IER5, MYC, PLK2, SESN1, TNFSF4, ZMAT3
Meadows et al. 2010. [55]	6 female C57BL/6 mice per group	X-Ray	PBI to AH, PH or HL	PBMCs	0.5	1.49 (AH & PH) 1.25 (HL)	6 hours	Custom mouse microarrays using Operon’s Mouse Genome Oligo set (v. 4.0)	Selected genes available in S.M.	NS	CCNG1, CDKN1A
					2						
					10						
Paul et al. 2014. [56]	8 male C57BL/6 mice for each time	^137^Cs	Internal emitter	WB in PAXgene RNA tubes	2	NS	2	Agilent Whole Mouse Genome Microarrays 4X44K v2 (G4846A)	GEO database: GSE52690	619	AEN, ASCC3, BAX, BBC3, FBXO22, FDXR, MDM2, PCNA, PHPT1, RPS27L, SESN1, TRIAP1, XPC
					2.7		3			1493	
					4.1		5			466	
					9.5		20			6375	
					9.9		30			6213	
Paul et al. 2015. [57]	6 male C57BL/6 mice per dose and dose/rate	X-Ray	TBI	WB in PAXgene RNA tubes	1.1, 2.2 & 4.4	0.00309	24 hours	Agilent Whole Mouse Genome Microarrays 4X44K v2 (G4846A)	GEO database: GSE62623	922	AEN, BAX, CCNG1, CDKN1A, EI24, PLK2, TRIAP1, ZMAT3
						1.03				869	

AH: anterior hemibody; GEO, Gene Expression Omnibus; HL: hind limb; NS: non specified; PBI: partial body irradiation; PBMC: peripheral blood mononuclear cell; PH: posterior hemibody; S.M., Supplementary Material; TBI: total body irradiation; WB, Whole Blood

Interestingly, none of the mouse studies used an ex-vivo irradiation approach but rather performed partial/total body irradiation or assessed the effect of an internal emitter, reflecting the greater flexibility of mouse models for such investigations. Genes in the mouse blood show a similar response to genes from human blood since 26 genes are in common with the 33 human genes panel identified in this study. These results indicate that the mouse model may be useful in extrapolating to a human radiation response. However, dose-response is not always correlated between species, probably due to some species-specific metabolism or to differences in experiment configuration (in vivo vs. ex vivo). Beyond the mouse model, and in order to be phylogenetically closer to human biology, a recent study used a non-human primate (NHP) model to compare the gene expression response with *ex-vivo* human blood exposed to a broad range of radiation doses [51]. The authors showed that a robust NHP biodosimetry model can be built using interspecies-correlated genes, and that, by using multiple regression-based cross-species conversion of expression values, absorbed dose in human samples can be accurately predicted by the NHP model.

## Discussion

Many studies have been performed using large-scale approaches to investigate radiation blood response and identify dosimetry biomarkers. However, due to complex logistics, these studies are limited either by the number of patients or by the number of different timing points or radiation doses, thus providing few data points for relevant direct comparisons. According to our selection criteria, we compiled in this systematic review 24 microarray studies, regrouping a total of 264 individuals, 21 different radiation doses and 13 different timing points for a total of 94 data points. Using this approach, we identified 31 genes showing differential expression in human blood between non-irradiated and irradiated samples, for at least one radiation dose in half or more of these studies. The aim of this review is to focus on the identification of reproducible radiation dosimetry biomarkers. This means the expression level of a gene candidate has to be significantly modified after IR but most importantly these changes have to be dose-dependent. Therefore, we performed a statistical analysis in order to highlight the genes with the highest correlation between expression level and radiation dose. The top 5 genes are: TNFSF4, TMEM30A, FDXR, ZMAT3 and AEN.

In order to assess the diagnostic ability of these genes for discriminating radiation doses < 2 Gy and ≥ 2 Gy, we also created ROCs. Results showed that TNFSF4, FDXR, MYC, ZMAT3 and GADD45A are the top 5 genes with the highest Youden’s index suggesting they have the most robust discriminating power. When sensitivity is fixed at 100% in order to decrease the number of false negatives, although results differed, TNFSF4, FDXR and ZMAT3 still have a good performance score. Although ZMAT3 has been less studied, TNFSF4 and FDXR are known to be radiation-responsive and are often used in candidate gene approaches and validation panels [58–63]. Recently, a study provided an in vivo dose-response of the FDXR gene, both for very low doses or partial body exposure, showing good correlation between physically and biologically assessed doses, thus confirming the great potential of this gene as candidate radiation dosimetry biomarker [64]. Moreover, these genes satisfied inter-laboratory comparisons that demonstrated that the dose estimates are always comparable, irrespective of the approach chosen by the different labs, suggesting that the results are independent from the protocol for preserving blood during incubation times [65].

Although these genes are promising individually, we next investigated combinations of gene biomarkers in order to improve their performance. As such, we showed that 12 different combinations of three genes display an AUC equal to 1 suggesting they are able to discriminate radiation doses < 2 Gy and ≥ 2 Gy with 100% accuracy. However, such a score has to be taken carefully. Even by compiling several studies, our panel is still small for large validation and we noticed that the best score was obtained with genes having few data points. This suggests that an increase of sample numbers is likely to decrease diagnostic accuracy and, therefore, a signature with a larger number of genes may be required to keep the same level of performance. Nevertheless, as radiation response is complex, this approach confirmed the need for combinations instead of individual biomarkers.

All the studies selected for this review used microarray and therefore, candidate gene have been identified using this approach. The successful implementation of DNA microarray technologies requires the development of methods and techniques for the fabrication of microarrays, the selection of probes to represent genes, the quantification of hybridization, and data analysis [66]. The probe set definition issue is particularly of critical importance, as it can dramatically influence the interpretation and understanding of expression data derived from microarray experiments [67]. As such, it is necessary to clearly know the probe sequence in order to be certain of which gene we identified, but especially which isoform, as several probes can be used for a same gene. Therefore, particular attention has to be taken in results interpretation and data extrapolation, thus highlighting the importance of validation step by using gold-standard method such as qRT-PCR. This also highlights the need to optimize the assay and probe design to the end platform that will be ultimately used during a real scenario.

If a good biodosimetry marker panel needs to be dose-dependent, conversely it should not be responsive to other stresses like chemical or biological agents. For example, sex and smoking status do not seem to influence the prediction of radiation dose [33], although the radiation response of some genes could be affected by sex [68]. Most of the studies in our survey have been performed on healthy individuals. However, Someya et al. showed that miR-99a overexpression by 93% or more after irradiation was associated with an elevated incidence of rectal bleeding, suggesting the radiation-induced overexpression of this biomarker could differ with individual radiosensitivity. This highlights the difficulty of defining a threshold as a unique reference for gene expression level and the necessity to combine several biomarkers assays. Ideally, a biodosimetry signature should be a universal panel with stable expression throughout the time of interest, say 1–7 days. Our results showed the expected variations in gene response according to time after exposure thus highlighting again the importance of choosing candidate dosimetry biomarkers taking such factors into account. For example, even if some genes do not display the best correlation, they should be preferentially selected and combined in a signature if they show prolonged temporal stability rather than a gene with better performance but not reliable over time. Moreover, each radiological disaster being different, radiation dosimetry markers must also be characterized for the detection of radiation dose from different particle types and from a broad range of energies from 10's of keV to 100's of MeV. Thus, a radiation dosimetry biomarker has to be radiation-responsive with different radiation qualities and dose rates. Studies on normal tissue and cell lines showed that diverse transcriptional programs in cellular response mechanisms, and involved in the development of normal tissue damage, may be differentially affected by high and low linear energy transfer (LET) radiation [69,70]. Such studies inevitably suggest that gene expression could be different in function of the radiation quality. We saw in this review that most of the genes respond to both X-rays (low LET) and particles (high LET) and that the fold-change observed in response to particle irradiation is generally greater than the same dose of X rays. However, the variability of response to different qualities of radiation still remain largely unknown [71] as few studies have deeply investigated the effects of particle radiation or different dose rates, and additional information is needed to understand the impact of these parameters on biomarkers. Therefore, current data do not allow extrapolation of results or claims that such biomarkers can be applied for all types of situation or stimuli. In many case of radiation exposure, radiation does not occur alone and modulation of gene expression can be influenced by several confounding factors. For example, gene expression changes observed during spaceflight may not be only due to IR but by microgravity, sleep deprivation, isolation, etc., [72]. Changes observed during radiation treatment may also be influenced by chemotherapy [73,74]. The majority of the studies included in this review analyzed gene expression in blood from healthy donors after ex-vivo irradiation and, therefore, exclude such variables. As such, additional studies are required to investigate the effects of such parameters in establishing a universal biodosimetry gene signature.

Gene biomarkers may be a reliable assay to assess absorbed radiation dose. However, due to the multiple approaches and different protocols, results are not always consistent, making it difficult to highlight the best candidate(s). Therefore, we have compiled results from several microarray studies in order to highlight the most promising gene biodosimetry markers in human blood. However, even with such an approach, results cannot be clearly extrapolated. Recently, a study demonstrated a new method for identifying radiation dosimetry biomarkers across independent studies by developing a meta-analysis using eleven microarray datasets [40]. The authors identify 29 gene biomarkers for predicting high (>8Gy) and low (<2Gy) radiation exposure. Among these 29 biomarkers, only five genes are in common with our selection (AEN, FDXR, PLK2, RPS27L and SESN1) demonstrating the extreme data heterogeneity and the difficulty of analysis to provide a consistent biodosimetry signature. However, we did not focus our analysis on radiation doses superior to 8 Gy, and among the 51 biomarkers discriminating only the lower doses that they identified, fifteen genes are in common with our selection (AEN, CDKN1A, DDB2, FDXR, GADD45A, MDM2, MYC, PLK2, RPS27L, SESN1, TIGAR, TNFRSF10B, TNFSF4, TRIAP1 and TRIM22). In order to avoid selection bias, we chose to focus only on large-scale studies to identify promising radiation dosimetry biomarkers. Our literature search only identified microarray studies that fit our criteria. To our knowledge, no studies investigating gene expression after irradiation in human blood have so far used other large-scale approaches such as RNA-sequencing, although several studies have reported radiation-induced gene expression changes in cell lines by using RNA-seq approach [75,76]. Because of its high efficiency to determine the dynamics of the transcriptome compared to microarray approach [77], this method will probably continue to develop for the analysis of radiation-induced transcriptome changes in the next years.

Finally, this review focused on the expression of protein-coding genes in blood, but some studies also investigated other types of radiation biomarkers or other biofluids. Thus, gene expression changes in saliva have been assessed during radiation treatment for head and neck cancer [78]. Radiation-induced changes in non-coding RNAs such as miRNAs or recently discovered lncRNAs have also been investigated as potential radiation dosimetry biomarkers [79–84]. Since new “omics” approaches open the era of big data in biomarker discovery, large-scale proteomics studies have also been used to identify radiation biomarkers in blood [85,86], saliva [87] or even urine [88–90]. Metabolomics is also an emerging field to detect changes in metabolite expression during disease state or after stress induction. Thus, some metabolomics studies have investigated radiation-induced changes in metabolites expression in saliva [91] or urine [92–98] to provide an alternate approach for identifying new candidate biodosimetry markers. These different approaches pave the way to additional studies investigating the combination of several screening methods as a good alternative to study interactions between different molecular entities and highlight major pathways involved in radiation response in order to identify new radiation biomarkers.

## Supporting information

S1 PRISMA Checklist PRISMA guidelines checklist. (PDF)

S1 File Data extraction form. (PDF)

S2 File Guidelines for REMARK scores adapted for radiation biodosimetry studies. (PDF)

S1 Table Modified REMARK (mREMARK) scores breakdown for the 23 studies listed in Table 1. (PDF)

S2 Table Individual data synthesis of each 23 included studies. (PDF)

S3 Table Biological processes of the 33 protein-encoding genes after functional enrichments with String 10.5. (PDF)

S4 Table Genes classified according to their Spearman’s rank correlation coefficient (r) and p‐value between their normalized expression level and radiation dose across all 24 studies for early time ≤ 6 hours (Panel A) and late time ≥ 24 hours (Panel B) after exposure.
(PDF)

S5 Table AUC and diagnostic accuracies of the 33 genes discriminating radiation dose <2Gy and ≥2Gy classified according to their maximized Youden’s index at exposure time ≤6 hours (panel A) and ≥24 hours (panel B).
(PDF)

S6 Table Combinations of three genes that display area under the ROC curve (AUC) = 1 to discriminate radiation dose < 2 Gy from radiation dose ≥ 2 Gy. (PDF)

S1 Fig Spearman’s rank correlation of expression level of 31 genes, as represented by fold changes values and the radiation dose. (PDF)

S2 Fig Receiver operating characteristic (ROC) curve analysis of the 31 selected genes to discriminate radiation dose < 2Gy from radiation dose ≥2Gy. (PDF)

S3 Fig Receiver operating characteristic (ROC) curve analysis of combination of two genes that display area under the ROC curve (AUC) ≥ 0.95 to discriminate radiation dose < 2 Gy from radiation dose ≥ 2 Gy. (PDF)
