# Supplementary material for: Candidate gene biodosimetry markers of exposure to external ionizing radiation in human blood: A systematic review
Source: PLoS One. 2018 Jun 7;13(6):e0198851. doi: 10.1371/journal.pone.0198851 (PMC5991767; doi:10.1371/journal.pone.0198851)
Supplement: S1 File — (PDF) [file pone.0198851.s002.pdf]

Data extraction form

Reviewer:

First Author:

Year of Publication:

1. <u>Study design and sample collection</u>

What is the study design?

- ◻ Ex-vivo irradiation
- ◻ Total-body irradiation (TBI)

What type of population has been recruited?

- ◻ Healthy individuals
- ◻ Patients

What is the total number of participants?

n = ______ participants

n = ______ male n = ______ female

Which type of blood samples have been irradiated?

- ◻ Whole blood
- ◻ White blood cells
- ◻ Peripheral blood mononuclear cells (PBMCs)
- ◻ Specific cell type: __________

What type of anticoagulant has been used to collect whole blood samples?

- ◻ Heparin
- ◻ Sodium citrate
- ◻ EDTA
- ◻ Others: __________
- ◻ None (TBI)

What type of medium has been used to dilute/culture blood samples?

Medium: __________
Dilution: __________

Which type of samples have been culutred after irradiation?

- ◻ Whole blood
- ◻ White blood cells
- ◻ Peripheral blood mononuclear cells (PBMCs)
- ◻ Specific cell type: __________

What type of medium has been used to dilute/culture blood samples?

Medium: __________
Dilution: __________

2. Irradiation protocol

Radiation source: ___________________

Radiation dose(s): ____________________ Gy

Dose-rate(s): ____________________ Gy/min

When was RNA extraction after irradiation?

- ____________________ hours

3. Assay method

Which microarray (or any other large-scale approach) platform has been used?

- ____

Is data available on database?

- ◻ Yes
- ◻ No

If yes, database: __________ accession number: __________

Is full data available in supplementary data?

- □ Yes
- □ No

Has a validation step been included?

- □ Yes
- □ No

If yes, which has the approach been used? ________________

4. **Statistical analyses**

Are statistical analyses detailed in Material and Method?

- □ Yes
- □ No

What statistical analyses were employed?

- □ Comparative analyses
- □ Predictive analyses
- □ Other: ____

Was the statistical significance set at $p < 0.05$?

- □ Yes
- □ No

If no, cut-off: _______

5. **Results**

How many genes are differentially expressed at each radiation dose/timing point relative to control?

Dose: ______ Gy Time: ______ hours Numbers of genes: ______
Dose: ______ Gy Time: ______ hours Numbers of genes: ______
Dose: ______ Gy Time: ______ hours Numbers of genes: ______
Dose: ______ Gy Time: ______ hours Numbers of genes: ______
Dose: ______ Gy Time: ______ hours Numbers of genes: ______
Dose: ______ Gy Time: ______ hours Numbers of genes: ______
Dose: ______ Gy Time: ______ hours Numbers of genes: ______
Dose: ______ Gy Time: ______ hours Numbers of genes: ______

Dose: ______ Gy   Time: ______ hours   Numbers of genes: ______
Dose: ______ Gy   Time: ______ hours   Numbers of genes: ______

How many unique genes are differentially expressed for at least one radiation dose relative to control?

- _______ genes

______ upregulated genes   ______ down-regulated genes

List of genes (reported in excel file): _______________.xls
