# Supplementary material for: Candidate gene biodosimetry markers of exposure to external ionizing radiation in human blood: A systematic review
Source: PLoS One. 2018 Jun 7;13(6):e0198851. doi: 10.1371/journal.pone.0198851 (PMC5991767; doi:10.1371/journal.pone.0198851)
Supplement: S2 File — (PDF) [file pone.0198851.s003.pdf]

S2 File. Guidelines for REMARK scores adapted for radiation biodosimetry studies.

Introduction

1. State the marker examined, the rationale, the study objectives, and any pre-specified hypotheses.

Materials and Methods

Patients

2. Describe the characteristics of the study patients, including their source and inclusion and exclusion criteria.
3. Specify ethics committee, guideline and/or approval number

Specimen characteristics

4. Describe type of biological material used (including control samples) either for radiation and RNA extraction. Describe methods of preservation and storage.

Assay methods

5. Specify the radiation protocol, including details on radiation source, dosimetry, dose rate, beam characteristics and filtration for x-ray/linac sources and radiation setup. Statement of irradiator constancy measurements and output traceability to National Standards. References to written irradiation standards and/or protocols used.
6. Specify the assay method used and provide (or reference) a detailed protocol, including specific reagents or kits used, quality control procedures, reproducibility assessments, quantitation methods, and scoring and reporting protocols. Specify whether and how assays were performed blinded to the study endpoint.

Study design

7. State the method of case selection, including whether prospective or retrospective and whether stratification or matching (for example, by gender or age) was used. Specify the time period from which cases were taken, the end of the follow-up period, and the median follow-up time.
8. List all candidate variables initially examined or considered for inclusion/exclusion in models.
9. Give rationale for sample size; if the study was designed to detect a specified effect size, give the target power and effect size.

Statistical analysis methods

10. Specify all statistical methods, including details of any variable selection procedures and other model-building issues, how model assumptions were verified, and how missing data were handled.
11. Clarify how marker values were handled in the analyses; if relevant, describe methods used for cutpoint determination.

Results

Data

12. Describe the flow of patients through the study, including the number of patients included in each stage of the analysis (a diagram may be helpful) and reasons for dropout. Specifically, both overall and for each subgroup extensively

examined report the number of patients and the number of events.

13. For studies involving patients, report distributions of basic demographic characteristics (at least age and sex), standard (disease-specific) prognostic variables, including numbers of missing values.

Analysis and presentation

14. Show the relation of the marker to standard prognostic variables [radiation dose].
15. Present univariable analyses showing the relation between the marker and outcome, with the estimated effect (eg, hazard ratio and survival probability). Preferably provide similar analyses for all other variables being analyzed.
16. For key multivariable analyses, report estimated effects (eg, hazard ratio) with confidence intervals for the marker and, at least for the final model, all other variables in the model.
17. Among reported results, provide estimated effects with confidence intervals from an analysis in which the marker and standard prognostic variables [radiation dose] are included, regardless of their statistical significance.
18. If done, report results of further investigations, such as checking assumptions, sensitivity analyses, and internal validation.

Discussion

19. Interpret the results in the context of the pre-specified hypotheses and other relevant studies; include a discussion of limitations of the study.
20. Discuss implications for future research and clinical value.
