# Supplementary material for: Candidate gene biodosimetry markers of exposure to external ionizing radiation in human blood: A systematic review
Source: PLoS One. 2018 Jun 7;13(6):e0198851. doi: 10.1371/journal.pone.0198851 (PMC5991767; doi:10.1371/journal.pone.0198851)
Supplement: S1 Table — (PDF) [file pone.0198851.s004.pdf]

S1 Table. Modified REMARK (mREMARK) scores breakdown for the 24 studies listed in Table 1.

Studies	Total score	Criteria																			
		1	2	3	4	5	6	7	8	9	10	11	12	13	14	15	16	17	18	19	20
Beer et al. 2014	11	x	x	x	x	x	x	0	0	0	0	x	0	0	x	0	0	0	x	x	x
Broustas et al. 2017	12	x	x	x	x	x	x	0	0	0	x	x	0	0	x	x	0	0	x	x	x
Dressman et al. 2007	16	x	x	x	x	x	x	x	x	0	x	x	x	x	x	x	0	0	0	x	x
El-Saghire et al. 2013	10	x	x	x	x	x	x	0	0	0	0	x	0	0	0	0	0	0	x	x	x
Fachin et al. 2007	10	x	x	x	x	x	x	0	0	0	x	0	0	0	0	0	0	0	x	x	x
Ghandhi et al. 2015	13	x	x	x	x	x	x	0	0	0	x	x	0	0	x	0	0	x	x	x	x
Gruel et al. 2008	11	x	x	x	x	x	x	0	0	0	x	x	0	0	0	0	0	0	x	x	x
Henríquez Hernández et al. 2009	11	x	x	x	x	0	x	x	0	0	x	0	0	x	x	0	0	0	0	x	x
Kabacik et al. 2011	12	x	0	x	x	x	x	0	0	0	x	x	0	0	x	x	0	0	x	x	x
Knops et al. 2012	13	x	x	x	x	x	x	0	0	0	x	x	0	0	x	0	0	x	x	x	x
Macaeva et al. 2016	14	x	x	x	x	x	x	0	0	0	x	x	0	0	x	x	0	x	x	x	x
Mayer et al. 2011	12	x	x	x	x	x	x	x	0	0	x	0	0	x	x	0	0	0	0	x	x
Meadows et al. 2008	15	x	x	x	x	x	x	0	x	0	x	x	x	x	x	x	0	0	0	x	x
Nosel et al. 2013	12	x	x	x	x	x	x	0	0	0	x	x	0	0	x	x	0	0	0	x	x
Paul & Amundson. 2008	15	x	x	x	x	x	x	0	0	0	x	x	0	0	x	x	x	x	x	x	x
Paul & Amundson. 2011	19	x	x	x	x	x	x	x	x	0	x	x	x	x	x	x	x	x	x	x	x
Paul et al. 2011	16	x	x	x	x	x	x	0	x	0	x	x	0	x	x	0	x	x	x	x	x
Paul et al. 2013	15	x	x	x	x	x	x	0	0	0	x	x	0	0	x	x	x	x	x	x	x
Pogosova-Agadjanyan et al. 2011	16	x	x	x	x	x	x	0	0	0	x	x	0	x	x	x	x	x	x	x	x
Rouchka et al. 2016*	7	x	x	x	x	x	x	0	0	0	0	x	0	0	0	0	0	0	0	0	0
Templin et al. 2011	12	x	x	x	x	x	x	0	x	0	x	x	0	x	0	0	0	0	0	x	x
Versteyhe et al. 2013	11	x	x	x	x	x	x	0	0	0	x	x	0	0	x	0	0	0	0	x	x
Vinoth et al. 2014	9	x	x	0	x	x	x	0	0	0	x	0	0	0	x	0	0	0	0	x	x
Wen et al. 2011	13	x	x	0	x	x	x	0	0	0	x	x	0	x	x	x	0	0	x	x	x

* Rouchka et al. is a data paper
