# Supplementary material for: Candidate gene biodosimetry markers of exposure to external ionizing radiation in human blood: A systematic review
Source: PLoS One. 2018 Jun 7;13(6):e0198851. doi: 10.1371/journal.pone.0198851 (PMC5991767; doi:10.1371/journal.pone.0198851)
Supplement: S2 Table — (PDF) [file pone.0198851.s005.pdf]

S2 Table. Individual data synthesis of each 24 included studies

Study/Year	Dose (Gy)	Dose-rate (Gy/min)	Analysis time after IR (h)	Numbers of differentially expressed genes		Statistical analysis	Genes selected for qRT-PCR validation	Genes validated by qRT-PCR validation	REMARK score
				Down-regulated	Up-regulated				
Beer et al. 2014	60	NS	2	154	152	quantile normalization, only genes > the 40th percentile of the average expression of all samples were included	FDXR, GADD45A, PHPT1, SESN1, BBC3, NPTX1, GDF15, TUBA1C, p53, PASK, ARHGEF7, FASL, ZNF-80, PLEKHF1	FDXR, SESN1 at 20h; ZNF-80, PLEKHF1 at 4h	11
			4	1757	869				
			20	1904	2770				
Broustas et al. 2017	0.1	1.23	24	0	14	random-variance t test, p<0.001, FDR<5%	BAX, TNFRSF10B, ITLN2, AEN, VWCE, FDXR, PHLDA3, EDA2R	All genes validated at 0.1, 0.3, 0.5 and 1 Gy	12
	0.3			4	33				
	0.5			1	49				
	1			87	152				
	2			96	149				
	4			58	149				
Dressman et al. 2007	1.5-2	0.2	6	0	6	Benjamini & Hochberg, adjusted p<0.05 (via GEO2R analysis)	NA	NA	16
El-Saghire et al. 2013	0.05	0.03	8	104	80	paired samples t -test, p<0.05	PF4, GNG11, CCR4, POLH, DDB2, AEN, CDKN1A	PF4, GNG11, CCR4, POLH at 0.05 Gy; DDB2, AEN, CDKN1A, POLH at 1 Gy	10
	1			134	115				
Fachin et al. 2007	0.1	1.18	48	67	19	ANOVA to identify genes significantly (uncorrected p<0.10) modulated in the 4 groups then SAM method and pairwise comparison, FDR<5%, p<0.05	XAB2, RAD51L1	XAB2 at 0.1, 0.25 and 0.5Gy and RAD51L1 at 0.25Gy	10
	0.25			53	77				
	0.5			80	62				
Ghandhi et al. 2015	0.56	0.0031	24	22	49	random-variance t test, p<0.001, FDR<5%	AEN, APOBEC3H, FDXR, PHLDA3, CD70, DDB2, CDKN1A, GADD45A, ZMAT3, TNFSF9, MDM2, BAX, ASCC3, CCNG1, PVT1, RPS27L, BBC3, TNFRSF10B, TRIAP1, SESN1, PHPT1, XPC, POLH, ACTA2, FAS, TNFSF8, RAB7B, PPM1D, SLC4A11, F5, DRAM1, BTG3, MYC	All genes validated at 4.45 Gy for 0.0031 Gy/min and 1.03 Gy/min	13
	2.23			79	134				
	4.45			118	236				
	0.56	1.03		10	55				
	2.23			55	150				
	4.45			211	354				
Gruel et al. 2008	0.05	0.45	3	0	0	Benjamini & Hochberg correction, adjusted p<0.05 (via GEO2R analysis)	RPL19, RPS6, ATP5E, COX8A, NDUFA4, RPL21	RPL19, RPS6, ATP5E, COX8A, NDUFA4, RPL21 at 3h for CD4 cells after 0.05 and 0.5 Gy	11
	0.5			0	3				
	0.05		24	0	0				
	0.5			0	8				

Henríquez Hernández et al. 2009	2	1.5	24	30	51	Two-class paired test in SAM program, FDR < 10%	NA	NA	11
Kabacik et al. 2011	2	0.7	2	0	13	Paired 2-class SAM comparisons, FDR<5%	ATF3, BBC3, CCNG1, CDC25C, CDKN1A, DDB2, FDXR, GADD45A, MDM2, PHPT1, PLK3, PRC1, SESN1	All genes validated	12
	4			1	247				
	2		4	7	7				
	4			29	29				
Knops et al. 2012	0.02 & 0.1	0.0286	24	2	22	ANOVA, Benjamini & Hochberg correction, adjusted p<0.01 for medium and dose dose, adjusted p<0.05 for low dose to detect significant radiation-induced gene expression changes then Tukey's honestly significant difference test, p<0.05 to locate pairwise differences in gene expression between radiation doses	FDXR, PFKFB3, LY6G5C	FDXR for 0.02 Gy at 24h and for 0.1 Gy at 24 and 48h, PFKFB3 for 0.02 Gy and 0.1 Gy at 24h and 48h	13
			48	44	100				
	0.5 & 1	0.7	6	68	92				
			24	147	276				
			48	392	543				
	2 & 4		6	87	106				
			24	274	322				
			48	594	647				
Macaeva et al. 2016	0.1	0.26	8	17	108	Three-way ANOVA, FDR<5%	DDB2, POLH, MDM2, TNFRSF10B, FDXR, ASTN2, NDUFAF6, PCNA	DDB2, TNFRSF10B, FDXR for 0.1 and 1 Gy at 8 and 24h; ASTN2 for 1 Gy at 8 and 24h, MDM2, PCNA, POLH for 0.1 Gy at 8h and for 1 Gy at 8 and 24h	14
	1			14	111				
Mayer et al. 2011	5	0.575	6	63	90	paired samples t -test, adjusted p<0.05	NA	NA	12
Meadows et al. 2008	1.5-2	0.2	6	59	121	Benjamini & Hochberg correction, adjusted p<0.05 (via GEO2R analysis)	NA	NA	15
Nosel et al. 2013	0.005	0.05	2.5	275	500	Moderated t-test, Benjamini & Hochberg correction, adjusted p<0.05, FC>1.5	NA	NA	12
			5	257	480				
			7.5	85	275				
			10	34	150				
	0.01		2.5	149	240				
			5	89	337				
			7.5	174	361				
			10	217	421				
	0.025		2.5	177	312				
			5	142	410				
			7.5	94	372				
			10	143	421				

	0.05		2.5	178	388				
			5	48	274				
			7.5	90	430				
			10	111	209				
	0.1		2.5	270	448				
			5	69	206				
			7.5	113	406				
			10	65	534				
	0.5		2.5	389	529				
			5	149	287				
			7.5	51	194				
			10	296	465				
Paul & Amundson. 2008	0.5	0.82	6	125	204	random-variance F test, p<0.001, FDR<5%	CDKN1A, FDXR, PHPT1, BBC3, SESN1	All genes for all radiation doses	15
	2			116	213				
	5			114	215				
	8			114	215				
	0.5		24	31	104				
	2			34	101				
	5			34	101				
	8			34	101				
Paul & Amundson. 2011	0.1	0.82	6	75	214	random-variance F test, p<0.001, FDR<4%	DDB2, CDKN1A, FHL2, APOBEC3H	All genes for all radiation doses	19
	0.5			61	228				
	2			59	230				
Paul et al. 2011	1.25	0.1	4	219	473	random-variance F test, p<0.001, FDR<5%	CDKN1A, FDXR, BBC3, PHPT1, SESN1, DDB2, PCNA	All genes for all radiation doses	16
	3.75		24	920	668				
Paul et al. 2013	0.5	0.82	48	189	97	random-variance F test, p<0.001, FDR<5%	CDKN1A, DDB2, PCNA, PHPT1, GNLY, GZMA, NKG7	All genes for all radiation doses	15
	2			188	98				
	5			189	97				
	8			189	97				
Pogosova-Agadjanyan et al. 2011	0.15	7.6	3	113	219	Paired t-test, FC>1.2	CDKN1A, CDCA3, GADD45A, PSRC1, TNFSF4, KIF20A	CDCA3, GADD45A, TNFSF4 at 24h	16
			8	175	201				
			24	114	609				
	12		3	156	174				
			8	534	655				

			24	1159	803				
Rouchka et al. 2016	0.3	5.6	0.5	216	223	Limma at p ≤ 0.05	NA	NA	7
	1.5			238	165				
	3			292	202				
Templin et al. 2011	1.25	NS	4	129	168	random-variance t test, p<0.001	NA	NA	12
Versteyhe et al. 2013	1	2	4	0	23	SAM method, p<0.05	NA	NA	11
Vinoth et al. 2014	1	1.16	0.5	1117	298	Principal component analysis (PCA) and 1-way ANOVA, FC>1.5, p<0.05	NA	NA	9
Wen et al. 2011	4.5	0.045	24	268	237	RVM F-test, p<0.05, FDR<10%	DDB2, EI24, CDKN1A, CIZ1, PSMD14, SENP3, MAP1LC3A, ANKRD25, GZMA, EPS8, FASLG, HLA-DRB1	DDB2, EI24, CIZ1, PSMD14, SENP3, MAP1LC3A, ANKRD25, GZMA, EPS8, FASLG, HLA-DRB1 for all radiation doses	13
	9			277	228				
