# Supplementary material for: Candidate gene biodosimetry markers of exposure to external ionizing radiation in human blood: A systematic review
Source: PLoS One. 2018 Jun 7;13(6):e0198851. doi: 10.1371/journal.pone.0198851 (PMC5991767; doi:10.1371/journal.pone.0198851)
Supplement: S3 Table — (PDF) [file pone.0198851.s006.pdf]

S3 Table. Biological processes of the 33 protein-encoding genes after functional enrichments with String 10.5.

Pathway ID	Pathway description	Count in gene set	False discovery rate
GO:0006974	cellular response to DNA damage stimulus	15	1.70E-10
GO:0034644	cellular response to UV	8	1.70E-10
GO:0071478	cellular response to radiation	9	4.46E-09
GO:0071214	cellular response to abiotic stimulus	10	1.24E-08
GO:0033554	cellular response to stress	17	3.53E-08
GO:0009411	response to UV	7	1.10E-06
GO:0009314	response to radiation	10	1.63E-06
GO:0072331	signal transduction by p53 class mediator	7	1.63E-06
GO:0009628	response to abiotic stimulus	13	3.65E-06
GO:0043068	positive regulation of programmed cell death	10	7.49E-06
GO:0050896	response to stimulus	26	9.51E-06
GO:0072332	intrinsic apoptotic signaling pathway by p53 class mediator	5	9.85E-06
GO:0045786	negative regulation of cell cycle	9	1.51E-05
GO:0006915	apoptotic process	12	1.79E-05
GO:0006950	response to stress	19	1.79E-05
GO:0042981	regulation of apoptotic process	13	2.27E-05
GO:0010212	response to ionizing radiation	6	3.38E-05
GO:0043065	positive regulation of apoptotic process	9	5.03E-05
GO:0008630	intrinsic apoptotic signaling pathway in response to DNA damage	5	5.47E-05
GO:0042771	intrinsic apoptotic signaling pathway in response to DNA damage by p53 class mediator	4	6.61E-05
GO:0022402	cell cycle process	11	8.36E-05
GO:0007093	mitotic cell cycle checkpoint	6	9.92E-05
GO:0044773	mitotic DNA damage checkpoint	5	0.000212
GO:0048583	regulation of response to stimulus	17	0.00029
GO:0097190	apoptotic signaling pathway	7	0.000291
GO:0051726	regulation of cell cycle	10	0.000296
GO:0080135	regulation of cellular response to stress	9	0.0003
GO:0045862	positive regulation of proteolysis	7	0.000339
GO:0051716	cellular response to stimulus	22	0.000339
GO:1903047	mitotic cell cycle process	9	0.000349

GO:0048523	negative regulation of cellular process	18	0.000414
GO:0010604	positive regulation of macromolecule metabolic process	15	0.00042
GO:0007049	cell cycle	11	0.000587
GO:2001020	regulation of response to DNA damage stimulus	5	0.000652
GO:0000278	mitotic cell cycle	9	0.000734
GO:0065007	biological regulation	27	0.000756
GO:1902531	regulation of intracellular signal transduction	11	0.000756
GO:0019222	regulation of metabolic process	22	0.0008
GO:0080134	regulation of response to stress	11	0.000898
GO:0051052	regulation of DNA metabolic process	6	0.00107
GO:0060255	regulation of macromolecule metabolic process	20	0.00112
GO:0097193	intrinsic apoptotic signaling pathway	5	0.00131
GO:0048522	positive regulation of cellular process	18	0.00136
GO:0031571	mitotic G1 DNA damage checkpoint	4	0.0015
GO:0050789	regulation of biological process	26	0.00163
GO:0030162	regulation of proteolysis	8	0.00275
GO:0031325	positive regulation of cellular metabolic process	14	0.0037
GO:0043281	regulation of cysteine-type endopeptidase activity involved in apoptotic process	5	0.0037
GO:0050794	regulation of cellular process	25	0.0037
GO:0070059	intrinsic apoptotic signaling pathway in response to endoplasmic reticulum stress	3	0.00384
GO:0042770	signal transduction in response to DNA damage	4	0.0045
GO:0044763	single-organism cellular process	26	0.00597
GO:0035556	intracellular signal transduction	11	0.007
GO:0048518	positive regulation of biological process	18	0.00766
GO:0031323	regulation of cellular metabolic process	19	0.00782
GO:0043280	positive regulation of cysteine-type endopeptidase activity involved in apoptotic process	4	0.00782
GO:0090199	regulation of release of cytochrome c from mitochondria	3	0.00809
GO:0006290	pyrimidine dimer repair	2	0.0092
GO:1903896	positive regulation of IRE1-mediated unfolded protein response	2	0.0092
GO:0051054	positive regulation of DNA metabolic process	4	0.00981
GO:0007050	cell cycle arrest	4	0.0117
GO:0010332	response to gamma radiation	3	0.0117
GO:0032471	negative regulation of endoplasmic reticulum calcium ion concentration	2	0.0117
GO:0032270	positive regulation of cellular protein metabolic process	9	0.0128

GO:0070914	UV-damage excision repair	2	0.0141
GO:0080090	regulation of primary metabolic process	18	0.0166
GO:0010564	regulation of cell cycle process	6	0.0188
GO:0032075	positive regulation of nuclease activity	2	0.0198
GO:0006977	DNA damage response, signal transduction by p53 class mediator resulting in cell cycle arrest	3	0.022
GO:0060576	intestinal epithelial cell development	2	0.0225
GO:0032436	positive regulation of proteasomal ubiquitin-dependent protein catabolic process	3	0.0234
GO:0043066	negative regulation of apoptotic process	7	0.0259
GO:0071850	mitotic cell cycle arrest	2	0.0282
GO:0065009	regulation of molecular function	12	0.0302
GO:0010648	negative regulation of cell communication	8	0.0303
GO:0006289	nucleotide-excision repair	3	0.0307
GO:0042326	negative regulation of phosphorylation	5	0.0307
GO:0048584	positive regulation of response to stimulus	10	0.0326
GO:0048145	regulation of fibroblast proliferation	3	0.0335
GO:0010224	response to UV-B	2	0.034
GO:0060575	intestinal epithelial cell differentiation	2	0.034
GO:0070987	error-free translesion synthesis	2	0.0415
GO:0009966	regulation of signal transduction	11	0.0425
GO:0042493	response to drug	5	0.043
GO:0009615	response to virus	4	0.0442
GO:0010646	regulation of cell communication	12	0.0445
GO:0006281	DNA repair	5	0.0455
GO:0000718	nucleotide-excision repair, DNA damage removal	2	0.0467
GO:0032461	positive regulation of protein oligomerization	2	0.0467
GO:0035510	DNA dealkylation	2	0.0467
GO:0070230	positive regulation of lymphocyte apoptotic process	2	0.0467
GO:0097296	activation of cysteine-type endopeptidase activity involved in apoptotic signaling pathway	2	0.0467
GO:0006919	activation of cysteine-type endopeptidase activity involved in apoptotic process	3	0.0471
