# Supplementary material for: Candidate gene biodosimetry markers of exposure to external ionizing radiation in human blood: A systematic review
Source: PLoS One. 2018 Jun 7;13(6):e0198851. doi: 10.1371/journal.pone.0198851 (PMC5991767; doi:10.1371/journal.pone.0198851)
Supplement: S4 Table — Genes classified according to their Spearman’s rank correlation coefficient (r) and p‐value between their normalized expression level and radiation dose across all 24 studies for early time ≤ 6 hours (Panel A) and late time ≥ 24 hours (Panel B) after exposure. (PDF) [file pone.0198851.s007.pdf]

S4 Table. Genes classified according to their Spearman's rank correlation coefficient (r) and p-value between their normalized expression level and radiation dose across all 24 studies for early time ≤ 6 hours (Panel A) and late time ≥ 24 hours (Panel B) after exposure.

Panel A

Gene	r	p-value
ZMAT3	0.742	0.0114
TNFSF4	0.6349	0.0299
TMEM30A	0.5524	0.0429
ACTA2	0.603	0.0529
FDXR	0.421	0.0574
TNFRSF10B	0.4139	0.0697
ZNF79	0.4762	0.0866
MDM2	0.4274	0.1129
TRIM22	0.5081	0.1134
AEN	0.3008	0.144
XPC	0.357	0.1458
PCNA	0.31	0.1714
CCNG1	0.3468	0.1716
MYC	-0.4236	0.1937
DDB2	0.2852	0.1983
EI24	0.3873	0.2132
FBXO22	0.3363	0.2375
SESN1	0.2911	0.2412
TRIAP1	0.2743	0.2418
GADD45A	0.2449	0.3122
PHPT1	0.2115	0.3996
ASCC3	0.2725	0.4122
IER5	0.2242	0.4365
CDKN1A	0.1905	0.4765
RPS27L	0.1578	0.5371
POLH	0.1529	0.5678
BBC3	0.1275	0.5819
CD70	0.129	0.6193
PLK2	0.06791	0.8594
TIGAR	-0.04216	0.8718
BAX	0.024	0.9199

Panel B

Gene	r	p-value
TNFSF4	0.8023	<0.0001
MDM2	0.6244	<0.0001
PLK2	0.6041	0.0002
TMEM30A	0.5811	0.0002
IER5	0.6881	0.0003
ZNF79	0.6106	0.0003
ZMAT3	0.5968	0.0004
GADD45A	0.5668	0.0005
TRIAP1	0.5552	0.0007
XPC	0.5312	0.0009
PCNA	0.5307	0.0009
FDXR	0.5528	0.001
ASCC3	0.5144	0.0011
PHPT1	0.5357	0.0016
CD70	0.5273	0.0016
AEN	0.518	0.002
SESN1	0.5567	0.0021
TNFRSF10B	0.5182	0.0024
ACTA2	0.5	0.0026
CDKN1A	0.5068	0.0031
RPS27L	0.4602	0.0047
TRIM22	0.4772	0.0057
FBXO22	0.5244	0.006
MYC	-0.5241	0.006
DDB2	0.4316	0.0061
POLH	0.4393	0.0065
CCNG1	0.4379	0.0096
BBC3	0.4205	0.0166
EI24	0.4958	0.0262
TIGAR	0.4951	0.0265
BAX	0.3108	0.0651
