# Supplementary material for: Candidate gene biodosimetry markers of exposure to external ionizing radiation in human blood: A systematic review
Source: PLoS One. 2018 Jun 7;13(6):e0198851. doi: 10.1371/journal.pone.0198851 (PMC5991767; doi:10.1371/journal.pone.0198851)
Supplement: S5 Table — AUC and diagnostic accuracies of the 33 genes discriminating radiation dose <2Gy and ≥2Gy classified according to their maximized Youden’s index at exposure time ≤6 hours (panel A) and ≥24 hours (panel B). (PDF) [file pone.0198851.s008.pdf]

S5 Table. AUC and diagnostic accuracies of the 33 genes discriminating radiation dose <2Gy and ≥2Gy classified according to their maximized Youden's index at exposure time ≤ 6 hours (panel A) and ≥ 24 hours (panel B).

Panel A

Gene	AUC [95% CI]	Sensitivity (%)	Specificity (%)	PPV (%)	NPV (%)	False positive (%)	False negative (%)	Youden's index
ZMAT3	0.933, [0.78-1.09]	100	83	83	100	17	0	0.83
ACTA2	0.833, [0.58-1.09]	75	100	100	60	0	40	0.75
TNFSF4	0.778, [0.49-1.07]	67	100	100	75	0	25	0.67
TNFRSF10B	0.691, [0.37-1.01]	67	93	80	87	20	13	0.6
XPC	0.650, [0.37-0.93]	60	100	100	67	0	33	0.6
ASCC3	0.607, [0.26-0.96]	57	100	100	57	0	43	0.57
EI24	0.633, [0.30-0.97]	57	100	100	63	0	38	0.57
PLK2	0.762, [0.45-1.07]	57	100	100	50	0	50	0.57
FDXR	0.711, [0.43-1.00]	50	100	100	83	0	17	0.5
MYC	0.583, [0.21-0.96]	50	100	100	63	0	38	0.5
TRIM22	0.733, [0.42-1.04]	50	100	100	63	0	38	0.5
ZNF79	0.708, [0.41-1.00]	50	100	100	73	0	27	0.5
AEN	0.610, [0.33-0.89]	50	94	80	80	20	20	0.44
GADD45A	0.522, [0.24-0.87]	40	100	100	60	0	40	0.4
SESN1	0.550, [0.27-0.83]	40	100	100	57	0	43	0.4
BBC3	0.546, [0.27-0.82]	56	83	71	71	29	29	0.39
CCNG1	0.576, [0.28-0.87]	38	100	100	64	0	36	0.38
FBXO22	0.625, [0.32-0.93]	38	100	100	55	0	45	0.38
MDM2	0.625, [0.32-0.93]	38	100	100	58	0	42	0.38
PHPT1	0.525, [0.21-0.84]	38	100	100	67	0	33	0.38
POLH	0.531, [0.21-0.85]	63	75	71	67	29	33	0.38
CD70	0.514, [0.17-0.86]	57	80	67	73	33	27	0.37
PCNA	0.600, [0.34-0.85]	36	100	100	59	0	41	0.36
DDB2	0.558, [0.29-0.83]	60	75	67	69	33	31	0.35
RPS27L	0.531, [0.24-0.82]	33	100	100	60	0	40	0.33
BAX	0.545, [0.14-0.82]	70	60	64	67	36	33	0.3
TRIAP1	0.550, [0.28-0.82]	30	100	100	59	0	41	0.3
IER5	0.552, [0.20-0.90]	67	63	57	71	43	29	0.29
TMEM30A	0.633, [0.33-0.94]	71	57	63	67	38	33	0.29
TIGAR	0.570, [0.28-0.86]	50	78	67	64	33	36	0.28
CDKN1A	0.556, [0.26-0.85]	22	100	100	50	0	50	0.22

Panel B

Gene	AUC [95% CI]	Sensitivity (%)	Specificity (%)	PPV (%)	NPV (%)	False positive (%)	False negative (%)	Youden's index
TNFSF4	0.904, [0.80-1.00]	72	100	100	77	0	23	0.72
TRIAP1	0.771, [0.60-0.94]	70	100	100	70	0	30	0.7
IER5	0.813, [0.63-0.99]	69	100	100	58	0	42	0.69
MYC	0.767, [0.59-0.95]	68	100	100	54	0	46	0.68
FDXR	0.810, [0.65-0.97]	72	93	93	72	7	28	0.65
GADD45A	0.761, [0.59-0.93]	65	100	100	67	0	33	0.65
PLK2	0.785, [0.63-0.94]	65	100	100	65	0	35	0.65
RPS27L	0.728, [0.55-0.91]	65	100	100	70	0	30	0.65

TMEM30A	0.765, [0.60-0.93]	65	100	100	68	0	32	0.65
ZMAT3	0.803, [0.64-0.96]	65	100	100	70	0	30	0.65
ZNF79	0.819, [0.67-0.97]	65	100	100	70	0	30	0.65
MDM2	0.789, [0.64-0.94]	62	100	100	64	0	36	0.62
PCNA	0.748, [0.58-0.91]	62	100	100	65	0	35	0.62
PHPT1	0.756, [0.58-0.93]	70	92	93	65	7	35	0.62
CD70	0.764, [0.60-0.93]	60	100	100	62	0	38	0.6
CDKN1A	0.738, [0.56-0.91]	60	100	100	60	0	40	0.6
TRIM22	0.747, [0.56-0.93]	60	100	100	74	0	26	0.6
DDB2	0.709, [0.54-0.88]	64	94	93	67	7	33	0.58
EI24	0.730, [0.51-0.95]	58	100	100	62	0	38	0.58
CCNG1	0.717, [0.54-0.90]	56	100	100	67	0	33	0.56
TNFRSF10B	0.762, [0.59-0.93]	75	81	80	76	20	24	0.56
XPC	0.750, [0.58-0.92]	56	100	100	69	0	31	0.56
ACTA2	0.745, [0.58-0.91]	55	100	100	61	0	39	0.55
ASCC3	0.746, [0.58-0.91]	60	94	92	67	8	33	0.54
BBC3	0.698, [0.51-0.89]	61	93	92	65	8	35	0.54
FBXO22	0.757, [0.57-0.94]	67	88	92	54	8	46	0.54
AEN	0.767, [0.60-0.93]	67	87	86	68	14	32	0.53
SESN1	0.780, [0.61-0.95]	53	100	100	65	0	35	0.53
POLH	0.724, [0.56-0.89]	50	100	100	63	0	37	0.5
TIGAR	0.722, [0.49-0.96]	82	67	75	75	25	25	0.48
BAX	0.613, [0.43-0.80]	30	100	100	53	0	47	0.3
