# Supplementary material for: Candidate gene biodosimetry markers of exposure to external ionizing radiation in human blood: A systematic review
Source: PLoS One. 2018 Jun 7;13(6):e0198851. doi: 10.1371/journal.pone.0198851 (PMC5991767; doi:10.1371/journal.pone.0198851)
Supplement: S6 Table — (PDF) [file pone.0198851.s009.pdf]

S6 Table. Combinations of three genes that display area under the ROC curve (AUC) = 1 to discriminate radiation dose < 2 Gy from radiation dose ≥ 2 Gy.

AEN + EI24 + IER5
BAX + IER5 + TNFSF4
EI24 + IER5 + TNFSF4
EI24 + IER5 + ZMAT3
FBXO22 + IER5 + TNFSF4
FDXR + IER5 + MYC
FDXR + IER5 + SESN1
FDXR + IER5 + TNFSF4
IER5 + PHPT1 + TNFSF4
IER5 + RPS27L + ZMAT3
IER5 + TIGAR + TNFSF4
IER5 + TNFSF4 + TRIAP1
