# Supplementary material for: Candidate gene biodosimetry markers of exposure to external ionizing radiation in human blood: A systematic review
Source: PLoS One. 2018 Jun 7;13(6):e0198851. doi: 10.1371/journal.pone.0198851 (PMC5991767; doi:10.1371/journal.pone.0198851)

S1 Fig. Spearman's rank correlation of expression level of 31 genes, as represented by fold changes values and the radiation dose.

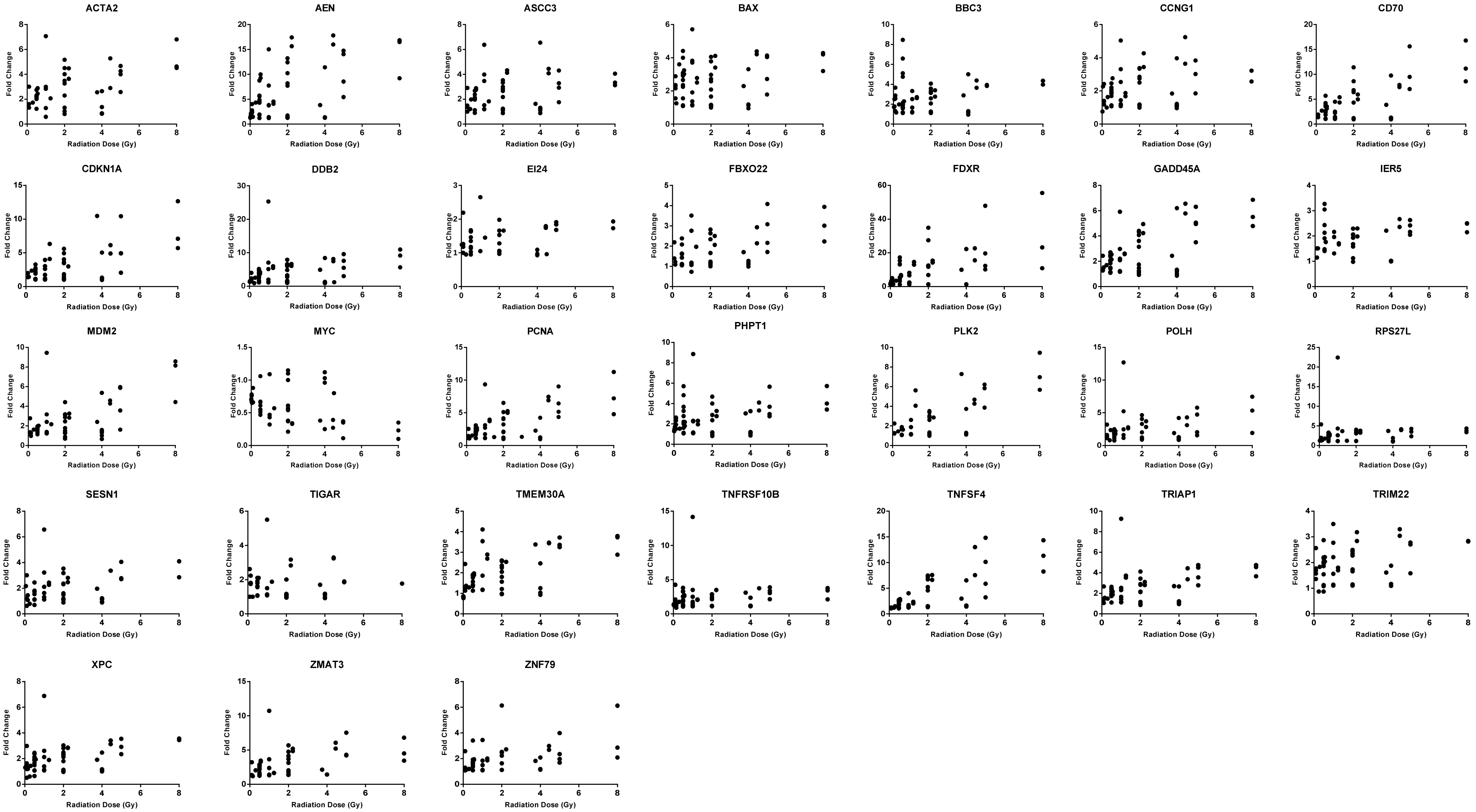

Supplement: S1 Fig — (PDF) [file pone.0198851.s010.pdf]
