# Supplementary material for: Candidate gene biodosimetry markers of exposure to external ionizing radiation in human blood: A systematic review
Source: PLoS One. 2018 Jun 7;13(6):e0198851. doi: 10.1371/journal.pone.0198851 (PMC5991767; doi:10.1371/journal.pone.0198851)

S2 Fig. Receiver operating characteristic (ROC) curve analysis of the 31 selected genes to discriminate radiation dose < 2Gy from radiation dose ≥2Gy.

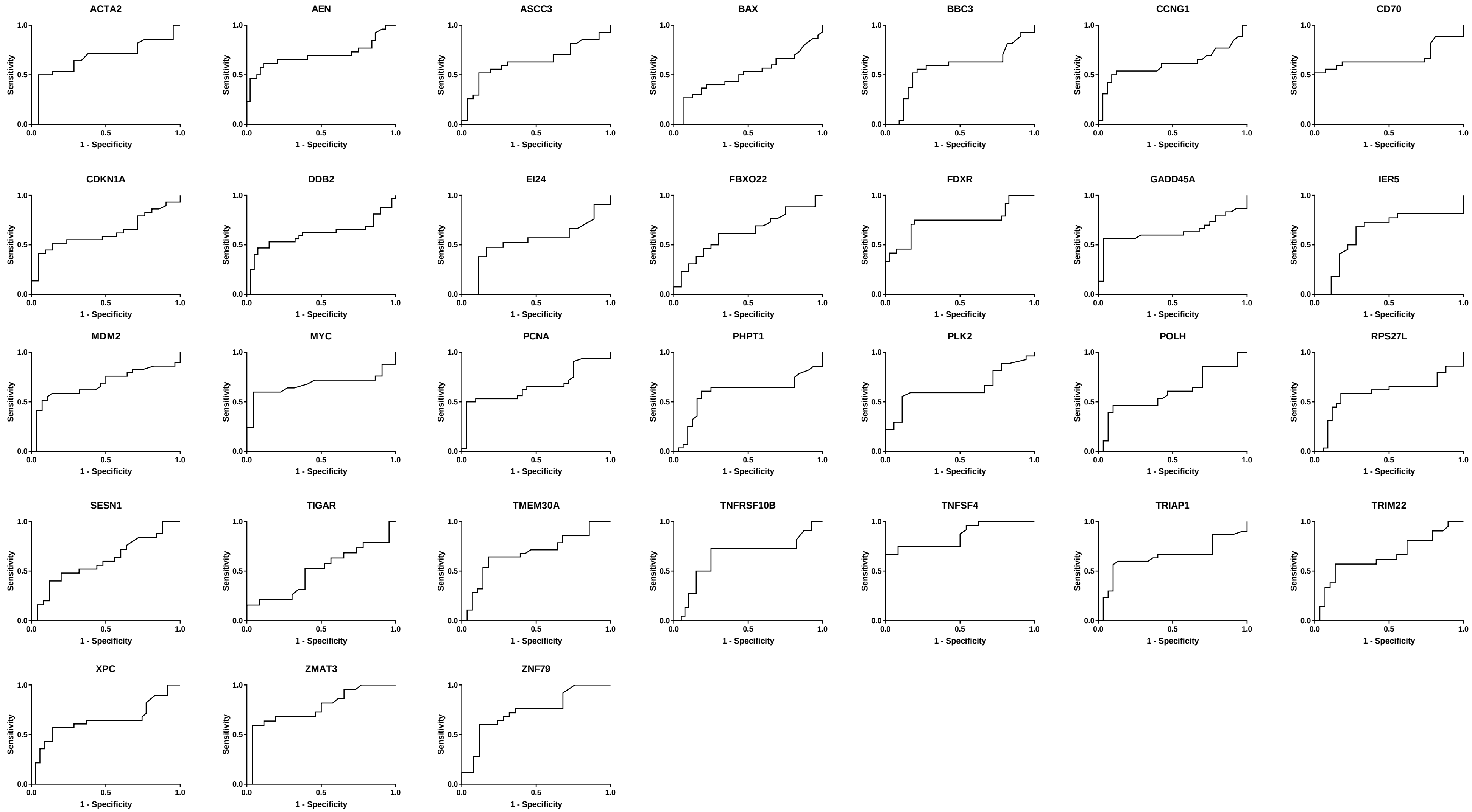

Supplement: S2 Fig — (PDF) [file pone.0198851.s011.pdf]
