# Supplementary material for: Candidate gene biodosimetry markers of exposure to external ionizing radiation in human blood: A systematic review
Source: PLoS One. 2018 Jun 7;13(6):e0198851. doi: 10.1371/journal.pone.0198851 (PMC5991767; doi:10.1371/journal.pone.0198851)

S3 Fig. Receiver operating characteristic (ROC) curve analysis of combination of two genes that display area under the ROC curve (AUC) ≥ 0.95 to discriminate radiation dose < 2 Gy from radiation dose ≥ 2 Gy.

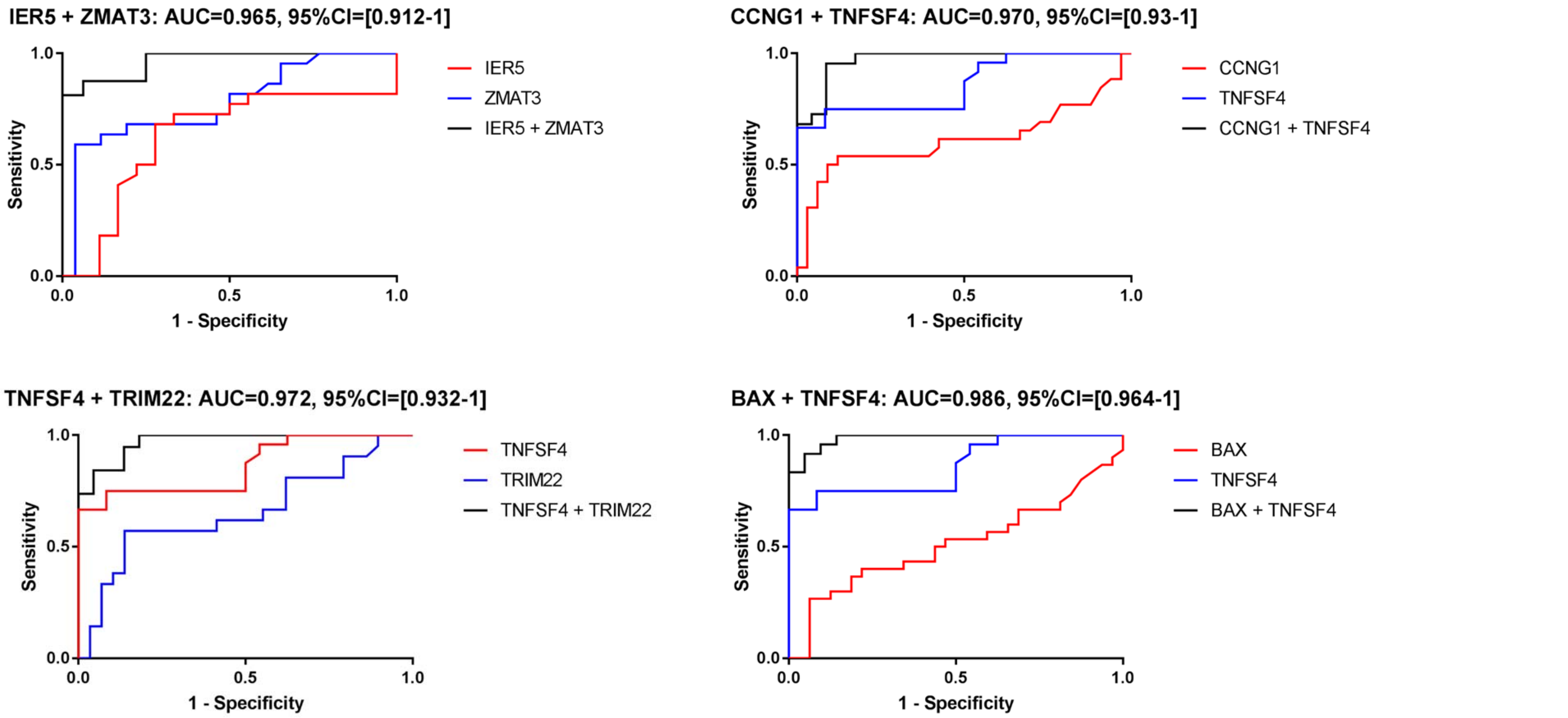

Supplement: S3 Fig — (PDF) [file pone.0198851.s012.pdf]
